# Signal peptide peptidase– and SPP-like 3–dependent shedding of α1,6-fucosyltransferase differentially affects core fucosylation

**DOI:** 10.1016/j.jbc.2026.111209

**Published:** 2026-01-29

**Authors:** Seita Tomida, Rebeca Kawahara, Kristina Mae Bienes, Yuko Tokoro, Takahiro Yamasaki, Yasuhiko Kizuka

**Affiliations:** 1 The United Graduate School of Agricultural Science, Gifu University, Gifu, Japan; 2 Institute for Glyco-Core Research (iGCORE), Nagoya University, Nagoya, Japan; 3 Institute for Glyco-Core Research (iGCORE), Gifu University, Gifu, Japan

**Keywords:** core fucose, FUT8, glycobiology, glycosylation, glycosyltransferase, *N*-linked glycosylation, SPP, SPPL3, shedding

## Abstract

Alpha1,6-fucosyltransferase (FUT8) biosynthesizes core fucose on *N*-glycans, which plays essential roles in various biological processes, including immunity and development. Although FUT8 is a Golgi-resident type II membrane protein, it is also secreted by an unknown mechanism. Here, we demonstrate that signal peptide peptidase (SPP) and signal peptide peptidase-like 3 (SPPL3), members of an intramembrane protease family, both cleave FUT8 for secretion. Knockout of SPP or SPPL3 in cells partially impaired FUT8 secretion, and double KO led to more drastic impairment in secretion, indicating that SPP and SPPL3 independently cleave FUT8. Sequencing analysis revealed that the *N* terminus of FUT8 in the media was mapped in the stem region, which is far from the expected cleavage site for SPP/SPPL3, suggesting that FUT8 undergoes two-step proteolytic processing, initially by SPP/SPPL3 and subsequently by another protease. Moreover, glycoproteomics suggested that the substrate glycoprotein preference of FUT8 was altered by knocking out SPP or SPPL3, highlighting the importance of FUT8 shedding in core fucosylation.

Core fucose is an α1,6-linked fucose residue attached to the reducing end GlcNAc in *N*-glycans. α1,6-Fucosyltransferase (FUT8) is known to be the sole enzyme responsible for catalyzing core fucosylation in mammals (Fig. 1*A*) (1, 2). The physiological importance of core fucosylation was demonstrated by analyses of various phenotypes of *Fut8* KO mice. Approximately 70% of *Fut8*-KO mice with the C57BL/6 background die within 3 days of birth, and the surviving mice exhibit growth retardation (3), enlarged alveoli (3), schizophrenia-like behaviors (4), and impaired T-cell functions (5, 6). Furthermore, core fucosylation has also been implicated in pathological conditions. In metastatic melanoma cells, core fucosylation is increased, and FUT8 knockdown suppresses invasive ability and metastasis *in vivo* (7). Moreover, in COVID-19 patients with severe symptoms, IgG targeting the SARS-CoV-2 spike protein lacks a core fucose (8), potentially contributing to excessive cytokine release and increased disease severity. These findings showed that core fucosylation is important for both physiological and pathological processes, thus it is crucial that elucidating the mechanisms by which the level or activity of FUT8 is regulated in cells.

**Figure 1:**
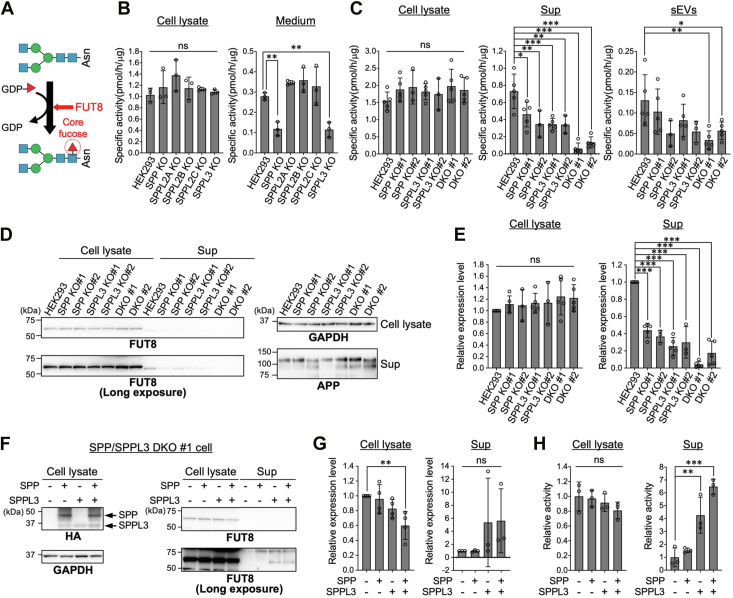
**Secretion of FUT8 depends on SPP and SPPL3**. *A*, schematic diagram of core fucosylation catalyzed by FUT8. Monosaccharide symbols are based on the Symbol Nomenclature for Glycans (SFNGs) (51). *B*, enzymatic activity of FUT8 was measured in both cell lysates and culture media of HEK293 WT cells and SPP/SPPL-family KO cells (*n* = 3, mean ± SD, ns: not significant, ∗∗: *p* < 0.01, Dunnett’s multiple comparison test). *C*, FUT8 activity was measured using cell lysates and soluble fractions (Sup) and membrane fractions (sEVs) from conditional media of HEK293 WT, SPP KO, SPPL3 KO, and SPP/SPPL3 DKO cells. #1 and #2 are two independent cell clones (HEK293, SPP KO#1, SPPL3 KO#1, DKO#,1 and DKO#2: *n* = 5, SPP KO#2, SPPL3 KO#2: *n* = 3, mean ± SD, ns: not significant, ∗: *p* < 0.05, ∗∗: *p* < 0.01, and ∗∗∗: *p* < 0.001, Dunnett’s multiple comparison test). *D*, Western blot analysis of proteins in the same samples used in (*C*). APP was used as a reference protein for the culture supernatant. *E*, quantification of the band intensity of FUT8 in (*D*). Band intensity was measured using ImageJ software (*n* = 5, mean ± SD, ns: not significant, ∗∗∗: *p* < 0.001, Dunnett’s multiple comparison test). *F*, SPP/SPPL3 DKO#1 cells were transfected with either 3 × HA-tagged SPP, SPPL3, or both, and proteins in the cell lysates and the soluble fraction of conditional media were analyzed by Western blotting. *G*, quantification of the band intensity of FUT8 in (*F*) (cell lysate: *n* = 4, Sup: *n* = 3, mean ± SD, ns: not significant, ∗∗: *p* < 0.01, Dunnett’s multiple comparison test). *H*, FUT8 enzymatic activity was measured in the same samples as in (*F*). The graph shows relative activity normalized to the activity in cells not expressing both SPP and SPPL3 (*n* = 3, mean ± SD, ns: not significant, ∗∗: *p* < 0.01, ∗∗∗: *p* < 0.001, Dunnett’s multiple comparison test). DKO, double knockou; FUT8, Alpha1,6-fucosyltransferase; SPP, signal peptide peptidase; SPPL3, signal peptide peptidase-like 3.

Previous studies have shown that intracellular activity and substrate preference of Golgi-resident glycosyltransferases are potentially regulated by their shedding into extracellular space. For instance, *N*-acetylglucosaminyltransferase-V (GnT-V, also designated MGAT5), which catalyzes the formation of the β1,6-GlcNAc branch on *N*-glycans, is cleaved and secreted (9, 10), and its intracellular level and activity are elevated upon inhibition of shedding (10, 11). Moreover, a secreted form of β-galactoside α2,6-sialyltransferase 1 (ST6GAL1), an enzyme responsible for terminal sialylation of *N*-glycans, sialylates soluble glycoproteins more efficiently than full-length ST6GAL1 (12). These findings showed the importance of glycosyltransferase shedding for shaping glycan profiles in cells. In addition, a number of enzymatically active soluble glycosyltransferases were reported to exist in extracellular fluids (9). Although the biological functions of many secreted glycosyltransferases remain poorly understood because of the scarcity of nucleotide sugar donors in extracellular fluids (13, 14), shedding of glycosyltransferases could be a factor for regulating the intracellular activity and level of glycosyltransferases.

FUT8 activity has also been detected in human blood, and its reduction is associated with the exacerbation of chronic obstructive pulmonary disease (15). This demonstrates that FUT8 is also shed while preserving the intact catalytic domain, similar to many other glycosyltransferases, and suggests that secretion of FUT8 is involved in pathogenesis in the lung. However, the molecular mechanism and physiological significance of FUT8 secretion remain largely unknown. Although a few proteases such as signal peptide peptidase-like 3 (SPPL3), β-site amyloid precursor protein (APP) cleaving enzyme 1, and furin have been shown to cleave other glycosyltransferases (9, 16, 17, 18, 19), it is unclear whether they also cleave FUT8. In particular, SPPL3 was identified as a major sheddase for dozens of glycosyltransferases (9). Knockout of SPPL3 was shown to result in dramatic glycan remodeling and critical alterations in immunity-related cell–cell interactions (20, 21, 22), highlighting the importance of SPPL3-mediated glycosyltransferase shedding. Notably, SPPL3 belongs to the intramembrane aspartyl protease family, comprising SPP, SPPL2A, SPPL2B, SPPL2C, and SPPL3 (23, 24). However, little is known about whether these proteases other than SPPL3 are involved in glycosyltransferase shedding.

In this study, we aimed to elucidate the mechanism and significance of FUT8 secretion by focusing on the SPP/SPPL3 family. Using KO cells deficient in each member of the SPP/SPPL family, we found that both SPP and SPPL3 are required for FUT8 secretion. Furthermore, glycoproteomic analysis suggested that the secretion of FUT8 affects its substrate protein preference. Our findings provide novel insights into the functional significance of glycosyltransferase secretion.

## Results

### SPP and SPPL3 are both required for FUT8 shedding

To identify the protease(s) required for FUT8 shedding, we focused on the SPP/SPPL family. This family comprises five multispanning proteases (SPP, SPPL2A, SPPL2B, SPPL2C, and SPPL3), all of which were reported to cleave transmembrane domains of membrane proteins or tail-anchored proteins (23). Notably, SPPL3 has been shown to cleave dozens of glycosyltransferases, such as GnT-V and B4GALT1 (9). However, it remains unclear whether other members also participate in the processing of glycosyltransferases. To examine whether any member of the SPP/SPPL family is involved in the shedding of FUT8, we generated HEK293 KO cells for *SPP* (*SPP* is also designated as *HM13*), *SPPL2A*, *SPPL2B*, and *SPPL2C* genes using the CRISPR–Cas9 system. Genotyping PCR and genomic sequencing confirmed disruption of each target gene in the KO cells (Fig. S1, *A* and *B*). We assessed *in vitro* FUT8 enzymatic activity of the cell lysates and culture media from WT and each KO cell line (Fig. 1*B*), using the established enzyme assay with a fluorescent *N*-glycan (25, 26). In the cell lysates, FUT8 activity remained largely unchanged in all KO cells compared with the level in WT (Figs. 1*B* and S1*C*, cell lysate). In contrast, in the media, SPP and SPPL3 KO samples showed reductions in activity to approximately 50% of the WT level, whereas FUT8 activity was not significantly changed in SPPL2A, SPPL2B, and SPPL2C KO compared with that in the WT (Figs. 1*B* and S1*C*, medium). This suggests that SPP and SPPL3 both contribute to FUT8 secretion.

Because FUT8 activity was reduced to roughly half of the WT level in the culture media of both SPP and SPPL3 KO cells (Fig. 1*B*, medium), we considered two possibilities: first, SPP and SPPL3 independently cleave FUT8 and both directly contribute to FUT8 secretion, or second, SPP or SPPL3 is required for the other’s protease activity in the same pathway for FUT8 cleavage. To test these possibilities, we generated a double-knockout (DKO) cell lacking both SPP and SPPL3 (Fig. S1*D*) and analyzed FUT8 secretion. Consistent with the results in Figure 1*B*, FUT8 activity in the cell lysates was only slightly increased by either single KO or DKO of SPP and SPPL3 (Figs. 1*C* and S2*A*, cell lysate). As culture media contain not only soluble proteins but also membrane components, including small extracellular vesicles (sEVs) in which FUT8 was detected in a previous study (27), we separated the soluble fraction (Sup) and the sEV fraction by ultracentrifugation, in each of which we measured FUT8 enzymatic activity (Fig. 1*C*). In the soluble fraction, FUT8 activity was reduced by approximately 50% in both SPP and SPPL3 single KO cells (Figs. 1*C* and S2*A*, Sup). Notably, in SPP/SPPL3 DKO cells, FUT8 activity was dramatically decreased, with DKO#1 showing less than 10% of the WT level (Figs. 1*C* and S2*A*, Sup). This additive effect demonstrates that SPP and SPPL3 are independently involved in FUT8 secretion. A similar trend was observed in the sEV fraction, in which FUT8 activity was modestly reduced in single KO cells and further decreased in DKO cells (Fig. 1*C*, sEVs). To simply examine the mechanism of FUT8 cleavage and secretion, we hereafter used the soluble fraction after removing sEVs by ultracentrifugation.

Next, we investigated the amount of FUT8 protein by Western blotting. Consistent with the enzymatic activity (Fig. 1*C*), the levels of FUT8 protein were not significantly different in the cell lysates between HEK293 WT and any KO cell line (Fig. 1, *D* and *E*). In contrast, FUT8 protein in the soluble fraction (Sup) was decreased in SPP KO and SPPL3 KO cell-derived Sup and further decreased in DKO (Fig. 1, *D* and *E*). APP, a control protein known to be secreted from HEK293 cells (28) was also included for the Western blotting analysis of Sup. We also confirmed that the expression levels of FUT8 mRNA were unchanged in SPP KO and SPPL3 KO cells compared with the level in WT cells (Fig. S2*B*). These results suggest that the enzymatic activity of FUT8 detected in Sup reflects the amount of secreted FUT8 protein, and that SPP and SPPL3 are both required for cleavage and secretion of FUT8.

To rule out the possibility that the reduced FUT8 secretion from DKO cells was due to an off-target effect, we performed a rescue experiment by overexpressing hemagglutinin (HA)-tagged SPP and SPPL3 in DKO cells (Fig. 1, *F*–*H*). Western blotting analysis confirmed the expression of SPP and SPPL3 (Fig. 1*F*, HA). Despite the similar polypeptide lengths (377 for SPP and 384 for SPPL3), SPP contains 2 *N*-glycosylation sites in its luminal regions, while SPPL3 was reported not to be *N*-glycosylated (10). The apparent higher molecular weight of SPP than SPPL3 (Fig. 1*F*), which is consistent with the previous reports (29, 30), could be derived from different modifications, such as glycosylation. Notably, the amount of FUT8 in Sup increased upon overexpression of the proteases, particularly SPPL3, although this difference was not statistically significant (Fig. 1*F*, FUT8, and G). Furthermore, FUT8 activity in Sup was increased with the expression of either SPP or SPPL3 alone, and more substantially with the coexpression of both SPP and SPPL3 (Figs. 1*H* and S2*C*). Together, these results demonstrate that SPP and SPPL3 directly contribute to FUT8 secretion.

**Figure 2:**
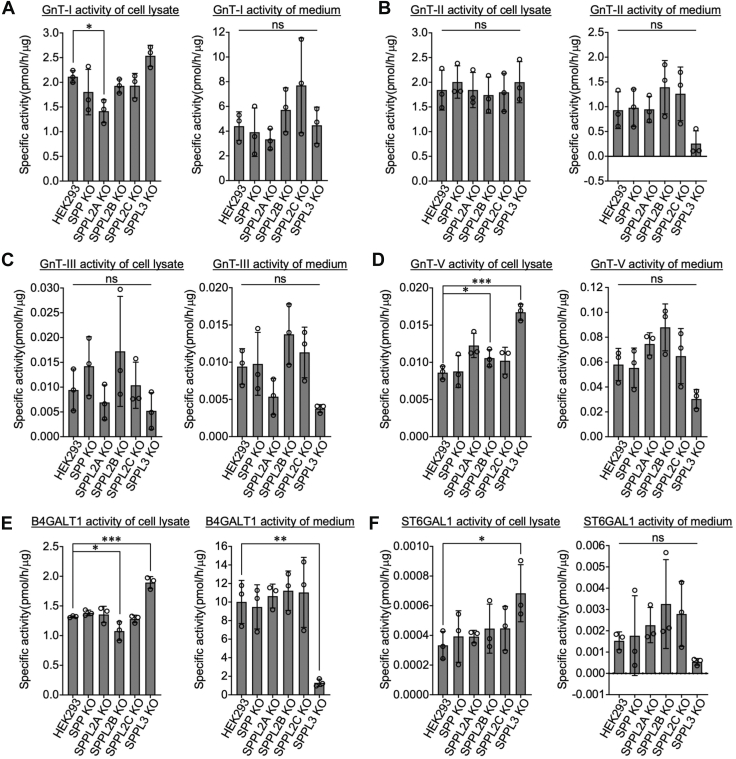
**Enzymatic activity of secreted glycosyltransferases**. Using the cell lysates and culture media, the *in vitro* activity of GnT-I (*A*), GnT-II (*B*), GnT-III (*C*), GnT-V (*D*), B4GALT1 (*E*), and ST6GAL1 (*F*) was assayed by incubating with their respective acceptor and donor substrates. The reaction products were separated and analyzed by HPLC, and the specific activity of each enzyme was quantified (*n* = 3, mean ± SD, ns: not significant, ∗: *p* < 0.05, ∗∗: *p* < 0.01, and ∗∗∗: *p* < 0.001, Dunnett’s multiple comparison test). FUT8, Alpha1,6-fucosyltransferase; GnT, GnT, *N*-acetylglucosaminyltransferase; ST6GAL1, β-galactoside α2,6-sialyltransferase.

To assess whether secretion mediated by the two proteases (SPP and SPPL3) is specific to FUT8 or more common to other glycosyltransferases, we next examined the secretion of other glycosyltransferases by measuring their enzymatic activity in cells and media (Figs. 2 and S3). Consistent with previous reports (9, 19), knocking out SPPL3 reduced the secretion of GnT-II (MGAT2), GnT-V (MGAT5), B4GALT1, and ST6GAL1 (Fig. 2, *B* and *D*–*F*). In contrast, KO of SPP did not affect the secretion of these glycosyltransferases (Fig. 2), unlike for FUT8. It should be noted that the specific activity of GnT-III, GnT-V, and ST6GAL1 was relatively low compared with the other enzymes, which could limit detection of statistical difference for these enzymes. These findings suggest that, while SPPL3 is required for the secretion of multiple glycosyltransferases, the secretory mechanism of each glycosyltransferase differs, and SPP is specifically involved in the cleavage and secretion of FUT8.

### Linker sequence in the stem region of FUT8 is essential for secretion

**Figure 3:**
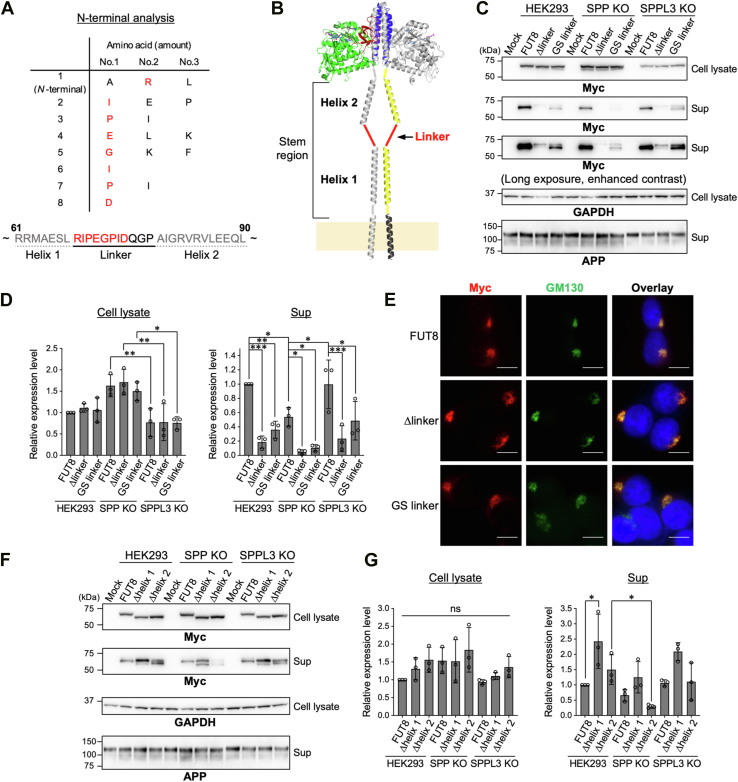
**Secretion of mutants in the stem region of FUT8**. *A*, *N*-terminal amino acid sequence of secreted FUT8 was analyzed by Edman degradation. The *horizontal axis* indicates amino acids detected in descending order of abundance, and the *vertical axis* shows the order of detection from the *N* terminus. Amino acids corresponding to the linker region of the FUT8 stem region are shown in *red*. Below the table, the amino acid sequence of the FUT8 stem region is shown. Residues identified by Edman degradation are shown in *red*. *B*, structural model of the FUT8 stem region, which consists of two α-helices (helix 1 and helix 2) connected by a linker. Individual domains are displayed in distinct colors: transmembrane (residues 7–31), *black*; stem region helix1 (residues 36–67) and helix2 (residues 79–105), *yellow*; catalytic domain, *green*; coiled-coil, *blue*; and SH3 domain, *red*. Note that this figure represents a model based on the determined crystal structure of FUT8 (PDB: 6VLD_B and 6VLD_C) with schematic stem and transmembrane regions. *C*, HEK293 cells, SPP KO#1 cells, and SPPL3 KO#1 cells were transfected with FUT8 WT, Δlinker, or GS linker. Cell lysates and soluble fractions (Sup) from the culture media were collected and analyzed by Western blotting. *D*, quantification of the band intensity of FUT8 in (*C*) (*n* = 3, mean ± SD, ∗: *p* < 0.05, ∗∗: *p* < 0.01, and ∗∗∗: *p* < 0.001, two-way ANOVA with *post hoc* Tukey’s multiple comparison test). Statistical results are presented only for intra–cell line comparison of the enzymes and inter–cell line comparison of each enzyme. *E*, immunofluorescence staining of HEK293 cells expressing Myc-tagged FUT8 WT, Δlinker, or GS linker (*red*: Myc, *green*: GM130, *blue*: DAPI, the scale bar represents 20 μm). *F*, HEK293 cells, SPP KO#1 cells, and SPPL3 KO#1 cells were transfected with FUT8 WT, Δhelix1, or Δhelix2. Cell lysates and soluble fractions from culture media were analyzed by Western blotting. *G*, quantification of the band intensity of FUT8 in (*F*) (*n* = 3, mean ± SD, ns: not significant, ∗: *p* < 0.05, two-way ANOVA with *post hoc* Tukey’s multiple comparison test). Statistical results are presented only for intra–cell line comparison of the enzymes and inter–cell line comparison of each enzyme. DAPI, 4′,6-diamidino-2-phenylindole; FUT8, Alpha1,6-fucosyltransferase; SPP, signal peptide peptidase; SPPL3, signal peptide peptidase-like 3.

To further elucidate the molecular mechanism of FUT8 secretion, we attempted to determine the *N*-terminal sequence of secreted FUT8. To prepare secreted FUT8, we overexpressed *C* terminally Myc/His-tagged full-length FUT8 in HEK293 WT cells, and secreted FUT8 with His-tag was purified from the media using Ni^2+^-beads (Fig. S4*A*). After excising the FUT8 band, the *N*-terminal amino acid sequence was analyzed by Edman degradation (Fig. S4*B*), demonstrating that the *N*-terminal sequence of secreted FUT8 is “RIPEGPID” in the stem region (Fig. 3*A*). The stem region of FUT8 consists of two helices, helix1 and helix2, which are connected through a linker (Fig. 3*B*) (31). The RIPEGPID sequence corresponds to the *N* terminus of the linker region, indicating that secreted FUT8 is cleaved between L67 and R68 within the stem region (Fig. 3*A*). However, the site did not meet the substrate specificity of SPP and SPPL3, since they are known to cleave substrates within or adjacent to their transmembrane domains (11, 19, 32, 33, 34). Therefore, we hypothesized that FUT8 secretion involves two-step cleavage: the first cleavage in the transmembrane region by SPP and SPPL3, followed by the second cleavage between helix1 and the linker region by another unknown protease.

To test this possibility, we generated two FUT8 mutants: one lacking the linker region (Δlinker) and the other in which the linker was replaced with the sequence GGGS (GS linker) (Fig. 3, *C* and *D*). We expected that Δlinker and GS linker mutants would undergo normal initial cleavage by SPP and SPPL3, but not the second cleavage. In the HEK293 cell lysates, the levels of the Δlinker and GS linker mutants were comparable to that of FUT8 WT (Fig. 3*C*, cell lysate, lanes 2–4 and 3D). However, contrary to our expectation, the amounts of Δlinker and GS linker proteins in Sup were greatly reduced compared with that of FUT8 WT (Fig. 3*C*, Sup, lanes 2–4 and *D*), suggesting that these mutants had defects in even the first cleavage. Moreover, in SPP KO cells, secretion of Δlinker and GS linker proteins was almost completely abolished, while their secretion in SPPL3 KO cells remained comparable to that in HEK293 WT cells (Fig. 3*C*, Sup, lanes 7–8 and 11–12 and 3*D*). This indicates that Δlinker and GS linker mutants primarily depend on SPP for the first cleavage. Additionally, in WT cells, the Δlinker migrated slower than FUT8 WT in Sup, and the GS linker mutant showed two distinct bands, with the upper band migrating above FUT8 WT (Fig. 3*C*, Sup, lanes 2–4). In contrast, in the cell lysates, the Δlinker and GS linker bands migrated slightly faster than FUT8 WT (Fig. 3*C*, cell lysate, lanes 2–4). These findings suggest that the upper bands of Δlinker and GS linker mutants in the media represent the products cleaved by SPP but not yet processed by the second protease, whereas the lower band of the GS linker mutant in the media corresponds to the fully processed form. Taken together, these results demonstrate that the linker sequence within the stem region is essential not only for the second cleavage step but also for the initial cleavage mediated by SPP.

To investigate the reason for the reduced secretion of the Δlinker and GS linker mutants, we examined their subcellular localization by immunofluorescence staining (Fig. 3*E*). Consistent with previous reports (31, 35), FUT8 WT colocalized well with the Golgi marker GM130. Similarly, both Δlinker and GS linker mutants also showed clear colocalization with GM130, indicating that these mutants are properly localized to the Golgi apparatus. This suggests that the reduced secretion of these mutants is not due to altered subcellular localization.

Next, we examined whether the helical parts (helix1 and helix2) in the stem region contribute to FUT8 secretion. Mutant FUT8 lacking either helix1 or helix2 (Δhelix1, Δhelix2) was expressed in cells, and its secretion was analyzed by Western blotting (Fig. 3, *F* and *G*). The overall secretion levels of Δhelix1 and Δhelix2 were not decreased compared with that of FUT8 WT in HEK293 cells, suggesting that these helices are not essential for the initial cleavage (Fig. 3*F*, lanes 3–4 and *G*). However, both mutants were detected as two bands in the supernatant, implying defects in the second cleavage. Intriguingly, secretion of Δhelix1 and Δhelix2 tended to decrease in SPP KO cells compared with WT cells, while KO of SPPL3 had minimal impact (Fig. 3*F*, lanes 7–8 and 11–12, and *G*). This suggests that these mutants also depend on SPP but not SPPL3 for the first cleavage.

### Specific residues in the transmembrane region of FUT8 governs SPP-dependent secretion of FUT8

**Figure 4:**
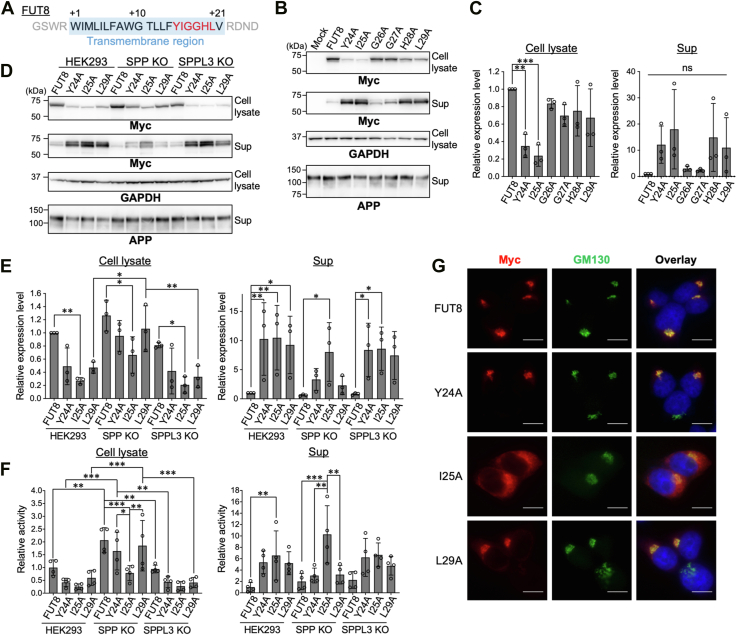
**Secretion of point mutants in the transmembrane region of FUT8**. *A*, amino acid sequence of the transmembrane region (TMR) of FUT8. The 10th tryptophan from the FUT8 *N* terminus is regarded as the first residue of TMR. The alanine-substituted residues are indicated in *red*. *B*, HEK293 cells were transfected with FUT8 WT or TMR point mutants. Cell lysates and soluble fractions (Sup) from the culture media were analyzed by Western blotting. *C*, quantification of the band intensity of FUT8 in (*B*) (*n* = 3, mean ± SD, ns: not significant, ∗∗: *p* < 0.01, ∗∗∗: *p* < 0.001, Dunnett’s multiple comparison test). *D*, HEK293 cells, SPP KO#1 cells, and SPPL3 KO#1 cells were transfected with FUT8 WT or TMR point mutants. Cell lysates and soluble fractions (Sup) from culture media were analyzed by Western blotting. *E*, quantification of the band intensity of FUT8 in (D) (*n* = 3, mean ± SD, ∗: *p* < 0.05, ∗∗: *p* < 0.01, two-way ANOVA with *post hoc* Tukey’s multiple comparison test). Statistical results are presented only for intra–cell line comparison of the enzymes and inter–cell line comparison of each enzyme. *F*, FUT8 enzymatic activity was measured in the cell lysates and soluble fractions (Sup) used in (*C*). The *graph* shows relative activity normalized to that of FUT8 WT in HEK293 cells (*n* = 4, mean ± SD, ∗: *p* < 0.05, ∗∗: *p* < 0.01, ∗∗∗: *p* < 0.001, and two-way ANOVA with *post hoc* Tukey’s multiple comparison test). Statistical results are presented only for intra–cell line comparison of the enzymes and inter–cell line comparison of each enzyme. *G*, immunofluorescence staining of HEK293 cells expressing Myc-tagged FUT8 WT, Y24A, I25A, or L29A (*red*: Myc, *Green*: GM130, *Blue*: DAPI, the scale bar represents 20 μm). DKO, double-knockout cell; FDR, false discovery rate; FUT8, Alpha1,6-fucosyltransferase; PhoSL, *Pholiota squarrosa* lectin; SPP, signal peptide peptidase; SPPL3, signal peptide peptidase-like 3.

To further explore the detailed mechanism of FUT8 cleavage by SPP and SPPL3, we focused on the transmembrane domain of FUT8. Previous studies have shown that SPP and SPPL3 cleave within the transmembrane region of their substrates, and that SPPL3 tends to cleave the *C*-terminal side of the transmembrane region of glycosyltransferases (19, 23). In addition, in the case of GnT-V, point mutation in the transmembrane region was shown to affect SPPL3-mediated cleavage (36). On the basis of these studies, we hypothesized that the sequence of the transmembrane region of FUT8 is critical for its cleavage by SPP and SPPL3. To verify this hypothesis, we generated alanine substitution mutants in which the 15th to 20th amino acid residues (YIGGHL) in the FUT8 transmembrane region were individually replaced with alanine (Fig. 4*A*). These mutants were expressed in HEK293 cells, and their levels in cell lysates and culture supernatants were examined (Fig. 4, *B* and *C*). The levels of G26A and G27A mutants were comparable to that of FUT8 WT in both cell lysate and Sup (Fig. 4*B*, cell lysate and Sup, lanes 2, 5–6 and *C*). In contrast, Y24A and I25A mutants exhibited markedly reduced intracellular levels (Fig. 4*B*; cell lysate, lanes 3–4 and 4C). In addition, the secretion levels of Y24A, I25A, H28A, and L29A mutants showed an increasing trend, although the differences did not reach statistical significance (Fig. 4*B*; Sup, lanes 3–4, 7–8 and 4C), suggesting that these residues play key roles in FUT8 cleavage. Although the mutated transmembrane region might serve as a signal peptide, prediction analysis using SignalP 6.0 indicated a low probability of acting as a signal peptide for all mutant transmembrane regions (Fig. S5). Based on this, we reason that the elevated trend in secretion of Y24A, I25A, H28A, and L29A is due to a change in either recognition of a FUT8 transmembrane region by SPP or SPPL3 or the subcellular localization of mutant FUT8.

To next investigate the dependence of the mutants on SPP and SPPL3, we expressed Y24A, I25A, and L29A in SPP KO and SPPL3 KO cells and examined their levels in cell lysate and Sup (Fig. 4, *D* and *E*). Compared with the level in HEK293 WT cells, the levels of Y24A and L29A mutants in Sup of SPP KO cells showed a decreasing tendency, but only minimal changes were observed in SPPL3 KO Sup (Fig. 4*D*, Sup, lanes 2–4, 6–8, 10–12 and 4E). Consistent with this, the enzymatic activity of Y24A and L29A was decreased in SPP KO Sup but barely reduced in SPPL3 KO Sup (Figs. 4*F* and S6). These results suggest that the cleavage of Y24A and L29A mutants largely depend on SPP. Meanwhile, the expression levels and the enzymatic activity of I25A in Sup were not impaired in SPP KO and SPPL3 KO (Figs. 4, *E* and *F*, and S6), suggesting that I25A may also be cleaved by a protease other than SPP and SPPL3.

As SPP and SPPL3 are mainly localized in the endoplasmic reticulum (ER) and Golgi apparatus, respectively (30, 37), the high dependence of Y24A and L29A mutants on SPP might be caused by a change in localization of the mutants. To test this possibility, we examined the subcellular localization of FUT8 Y24A, I25A, and L29A mutants by coimmunofluorescence staining with a Golgi marker GM130 (Fig. 4*G*). Both Y24A and L29A were localized mainly to the Golgi apparatus, whereas I25A was mainly localized to the Golgi but also showed partial ER-like localization. These findings suggest that the increased secretion of Y24A and L29A is not due to altered localization, but rather due to more efficient recognition of these mutants by SPP than that of FUT8 WT. In contrast, the altered localization of I25A may contribute to the enhanced cleavage by another protease. Taken together, these results demonstrate that specific amino acid residues in the transmembrane domain of FUT8, particularly Y24 and L29, play crucial roles in the cleavage by SPP and SPPL3.

### Secretion of FUT8 regulates core fucosylation

**Figure 5:**
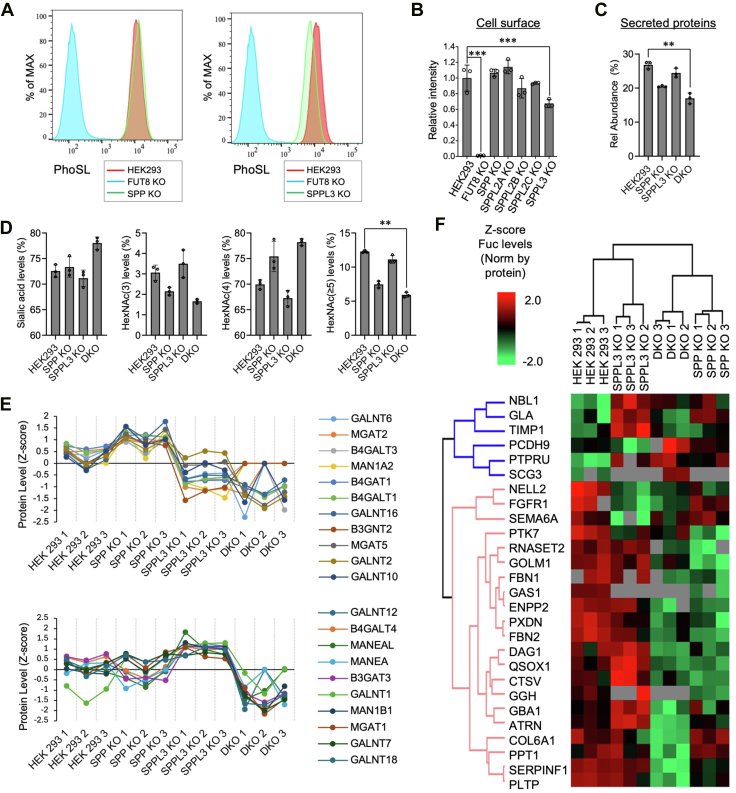
**Core fucose levels in SPP KO and SPPL3 KO cells**. *A*, HEK293 WT cells, FUT8 KO cells, SPP KO#1 cells, and SPPL3 KO#1 cells were stained with biotinylated PhoSL, followed by incubation with Alexa Fluor 488–conjugated streptavidin. Fluorescence intensity was analyzed by flow cytometry and visualized using FlowJo software. The horizontal axis represents fluorescence intensity and the vertical axis indicates the relative number of cells, normalized to the peak cell count in each sample. *B*, quantification of the FACS signal in (*A*). The core fucosylation level in HEK293 WT cells was set to 1.0 (*n* = 3, mean ± SD, ∗∗∗: *p* < 0.001, Dunnett’s multiple comparison test). *C*, relative abundance of fucosylated *N*-glycans in secreted proteins as determined by glycoproteomics. *D*, levels of *N*-glycan sialylation and branching in secreted proteins from WT and KO cells, based on glycoproteomics (data in *C* and *D*, *n* = 3, mean ± SD, ∗: *p* < 0.05, ∗∗: *p* < 0.01, and ∗∗∗: *p* < 0.001, Dunnett’s multiple comparison test). *E*, protein expression profiles of transmembrane glycosylation enzymes showing significant changes across HEK293, SPP KO, SPPL3 KO, and DKO cells (proteomics; *n* = 3 per group; one-way ANOVA with Benjamini–Hochberg correction; FDR < 0.05). *F*, heat map showing protein-specific fucosylation changes in SPP KO, SPPL3 KO, and DKO cells relatively to HEK293 control. Fucosylation levels were normalized to the corresponding protein abundance. Significant changes (ANOVA, followed by Benjamini–Hochberg correction, FDR <0.05) are indicated. DKO, double-knockout cell; FDR, false discovery rate; FUT8, Alpha1,6-fucosyltransferase; PhoSL, *Pholiota squarrosa* lectin; SPP, signal peptide peptidase; SPPL3, signal peptide peptidase-like 3.

To elucidate the biological significance of FUT8 secretion, we analyzed the levels of core fucose in SPP and SPPL3 KO cells. Based on a previous report showing that cleavage of another glycosyltransferase, ST6GAL1, enhances its activity toward soluble glycoproteins but not cell surface glycoproteins (12), we examined the levels of core fucosylation of the cell surface and secreted glycoproteins. First, the levels of core fucose on the cell surface were assessed by FACS with *Pholiota squarrosa* lectin (PhoSL) (38), which specifically binds to core fucose (Fig. 5, *A* and *B*). In SPP KO cells, the level of cell surface core fucose barely changed, while the level of core fucose in SPPL3 KO cells was significantly reduced (Fig. 5, *A* and *B*). This suggests that SPPL3-mediated secretion of FUT8 positively regulates core fucosylation of cell surface proteins.

Next, we performed glycoproteomics to examine the levels of fucosylation of secreted proteins (Table S1). Fucosylation of secreted proteins was significantly decreased in DKO cells. In comparison, SPP KO cells exhibited a modest but nonsignificant reduction, while SPPL3 KO cells showed only a slight decrease (Fig. 5*C*). Analysis of MS/MS spectra using GlycoDecipher to detect core fucosylation diagnostic ions further confirmed that the observed fucosylation changes were predominantly attributable to core fucose (Table S2). This suggests that SPP-mediated secretion of FUT8 has a larger impact on the core fucosylation of secreted proteins than SPPL3-mediated cleavage. Notably, glycoproteomic analysis of the secreted fraction revealed that SPP and SPPL3 modulate not only fucosylation but also sialylation and *N*-glycan branching, and the extents of these effects differ (Fig. 5*D*). For instance, DKO led to a slightly increased sialylation and a significant decrease in highly branching glycans with 5 or more HexNAc residues. Supporting this observation, proteomic profiling of secreted fractions demonstrated that SPP and SPPL3 modulate the cleavage and extracellular release of diverse proteins, notably including many Golgi-resident glyco-enzymes (Fig. 5*E* and Table S3), although we were not able to reliably detect FUT8 protein probably due to its low abundance. This proteomic profiling indicates that SPP- and SPPL3-mediated cleavage affects multiple glycosylation enzymes, suggesting broader consequences on glycosylation pathways beyond fucosylation. Notably, deletion of SPP and SPPL3 differentially altered FUT8 substrate specificity, as evidenced by distinct sets of proteins with markedly reduced fucosylation in SPP KO and SPPL3 KO cells (Fig. 5*F*). Collectively, these findings indicate that the fucosylation of the cell surface and secreted proteins is directly regulated by the distinct substrate specificities of FUT8 released through SPP- and SPPL3-mediated cleavage, as well as indirectly influenced by broader changes in the proteome and glycosylation pathways.

## Discussion

**Figure 6:**
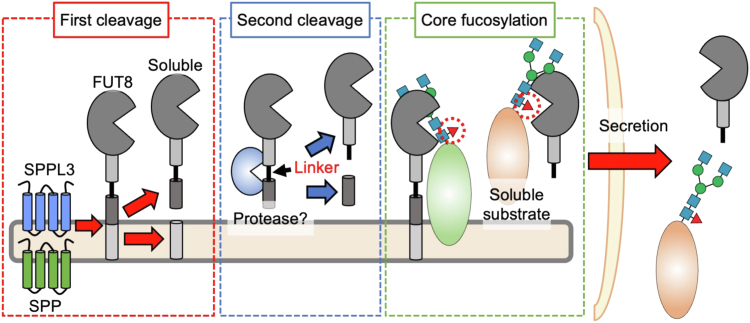
**Schematic model of FUT8 secretion and core fucosylation**. FUT8 is secreted extracellularly to outside cells through a two-step proteolytic process. In the first step, the transmembrane region of FUT8 is cleaved by SPP and SPPL3. In the second step, an unidentified protease cleaves the linker region of FUT8, resulting in the release of FUT8 into the extracellular space. It is possible that soluble FUT8 and membrane-bound FUT8 have different preferences for substrate glycoproteins. FUT8, Alpha1,6-fucosyltransferase.

The present study showed that FUT8 is secreted through a two-step proteolytic process, first by SPP and SPPL3 and subsequently by an unidentified protease. Furthermore, we found that blockade of FUT8 secretion resulted in less core fucosylation of both cell surface and secreted glycoproteins, with different impacts of SPP KO and SPPL3 KO. In particular, our glycoproteomic analysis of secreted proteins suggested that the substrate protein specificity of FUT8 was differentially affected by SPP KO and SPPL3 KO. Our findings provide novel insights into the regulation of core fucosylation by FUT8 secretion (Fig. 6).

By activity assay and Western blotting using cells with KO of SPP/SPPL family members, we demonstrated that SPP and SPPL3 are responsible for FUT8 secretion (Fig. 1, *B* and *C*). Previous studies showed that SPP, SPPL2A, and SPPL2B preferentially cleave substrates with short luminal domains, and SPP and SPPL2C favor tail-anchored proteins (23, 24). Since FUT8 has a large luminal domain (39, 40, 41), it is unlikely to be a typical substrate of these proteases other than SPPL3. However, SPP has also been shown to cleave XBP1u, which also possesses a long luminal domain, through interaction of SPP with ERAD-related factors such as Derlin1 and TRC8 (29), suggesting that these cofactors assist the substrate recognition of SPP. We showed that the luminal linker in the FUT8 stem region is also essential for the first SPP/SPPL3-mediated cleavage. However, SPP and SPPL3 themselves do not have long luminal domains. Therefore, it is possible that SPP and/or SPPL3 interact with accessory factors to recognize the linker region of FUT8. Our previous study has demonstrated that SPPL3-mediated cleavage of GnT-V depends on the *N*-glycan structure of GnT-V (10), suggesting a molecule interacting with SPPL3 for glycan-dependent recognition of substrates. However, because FUT8 lacks an *N*-glycosylation site, it is more likely that FUT8 is recognized by SPP/SPPL3 through a distinct mechanism from GnT-V. Additionally, SPPL2A KO showed a reduced tendency of GnT-III activity in the culture medium (Fig. 2), raising the possibility that SPPL2A recognizes GnT-III, an atypical substrate, by interacting with a cofactor. Identifying such cofactors of SPP/SPPLs could provide further insights into the mechanisms of glycosyltransferase secretion.

Interestingly, despite the reduction in secreted FUT8 in SPP and SPPL3 KO Sup, the intracellular FUT8 levels barely or only slightly changed for these KO cells (Fig. 1, *B*–*D*). Therefore, our data suggest that the steady-state levels of intracellular FUT8 are strictly regulated. This is also observed for other glycosyltransferases such as GnT-II and GnT-III, whose secretion is reduced in SPPL3 KO cells with no apparent changes in intracellular activity (Fig. 2). In contrast, the intracellular activity of GnT-V, B4GALT1, and ST6GAL1 are increased upon SPPL3 KO (Fig. 2) (10, 11), indicating the distinct dynamic mechanism for the rapid regulation of the level and activity of GnT-V, B4GALT1, and ST6GAL1 in cells. Future investigation into the degradation pathway and rate of FUT8 in SPP/SPPL3 KO cells should deepen our understanding of the mechanisms by which steady-state levels of cellular FUT8 are regulated.

Although the protease responsible for the second cleavage of FUT8 remains unknown, several characteristics can be inferred from the present findings and previous studies. Notably, a soluble form of FUT8 was originally purified from the culture medium of MKN45 cells for cDNA cloning and possessed the *N*-terminal amino acid, “RIPEG” (2). This sequence is fully consistent with our results shown in Figure 3*A*, suggesting existence of the conserved secretion mechanism of FUT8 involving the second protease. Since the second cleavage follows the action of Golgi-resident SPPL3 (30, 37), the second protease likely resides in the late Golgi or post-Golgi compartments. In addition, Δlinker, which is resistant to the first cleavage, also fails to undergo the second cleavage (Fig. 3*C*), and the level of secondary cleaved Δhelix1 was markedly reduced despite the presence of the linker (Fig. 3*F*). This suggests that not only the sequence but also the length of linker and helix1 (or distance from the membrane) is crucial for substrate recognition by the secondary protease. As the stem region does not affect FUT8 enzymatic activity itself (31), both cleavage products likely retain the catalytic function, although the biological role of the second cleavage remains unclear. Identifying the second protease would significantly advance our understanding of the molecular mechanism of FUT8 secretion.

A previous study on substrate recognition by SPPL3 showed that replacing the glycine residues at the 9th and 13th positions in the transmembrane region of GnT-V with proline increases SPPL3-mediated secretion of GnT-V by enhancing the flexibility of the α-helix in the transmembrane region (36). In contrast, replacement with leucine stabilizes the α-helix and suppresses the secretion of GnT-V (36). In our study, the secretion of FUT8 Y24A and L29A was increased compared with that of FUT8 WT (Fig. 4, *B*–*F*). Although the mutated sites differ from GnT-V, it is possible that the alanine substitutions at Y24 and L29 altered the flexibility of the transmembrane region of FUT8. Further structural studies of the transmembrane region of FUT8 could advance our understanding of the substrate specificity of SPP and SPPL3.

Our findings suggest that the secretion of FUT8 impacted the core fucosylation of proteins. Specifically, SPPL3 KO cells exhibited reduced core fucosylation of cell surface proteins, whereas SPP KO cells did so for secreted proteins. These results imply that the specificity of FUT8 toward glycoprotein substrates is altered by cleavage. As a possible mechanism behind this, accessibility of substrates could differ between membrane-bound and secreted forms of FUT8. The full-length FUT8 is anchored to the membrane, potentially restricting the access of its catalytic domain to certain substrates. In contrast, the secreted form, which is free from membrane constraints, might more easily access glycoprotein substrates. This possibility is supported by a previous study showing that soluble ST6GAL1 enhances sialylation of a soluble protein more effectively than does full-length ST6GAL1 (12).

In addition, SPP and SPPL3 are localized to the ER and the Golgi apparatus, respectively (30, 37). Therefore, FUT8 is likely cleaved by SPP in the ER and by SPPL3 in the Golgi. Thus, FUT8 cleaved at an early stage by SPP in the ER may follow a different trafficking route from FUT8 that is cleaved by SPPL3 after it reaches the Golgi. If these distinct trafficking routes expose FUT8 to different sets of glycoprotein substrates, it could explain the distinct substrate-specific effects of SPP- and SPPL3-mediated cleavage. Further investigation into the subcellular localization of FUT8 in SPP KO and SPPL3 KO cells will be a key to clarifying how secreted FUT8 regulates core fucosylation.

## Experimental procedures

### Reagents

The following antibodies were used: mouse anti-GAPDH (Merck Millipore, MAB374), mouse anti-APP (Merck Millipore MAB348), rabbit anti-HA tag (Cell Signaling Technology, 3724), mouse anti-Myc tag (Merck Millipore, 05–724), horseradish peroxidase (HRP)-anti-mouse IgG (GE Healthcare, NA931), HRP-anti-rabbit IgG (GE Healthcare, NA934), rabbit anti-GM130 (Cell Signaling Technology, 12480), Alexa546-anti-mouse IgG (Invitrogen, A10036), and Alexa488-anti-rabbit IgG (Invitrogen, A21206). Mouse anti-FUT8 (Fujirebio, clone 15C6) was kindly provided by Dr Eiji Miyoshi (Osaka University). Biotinylated PhoSL was kindly provided by J-Oil Mills.

### Cell culture

HEK293 cells were purchased from American type culture collection. HEK293 SPPL3 KO cells had already been established (10). HEK293 FUT8 KO cells were kindly provided by Dr Jianguo Gu (Tohoku Medical and Pharmaceutical University) (42). Cells were grown in Dulbecco’s modified Eagle’s medium supplemented with 10% fetal bovine serum and 50 μg/ml kanamycin at 37 °C under 5% CO_2_ conditions. To prepare proteins from medium, 6.0 × 10^6^ cells were cultured on a 15-cm dish. After 24 h of incubation, medium was removed and the cells were washed three times with PBS. Subsequently, the cells were cultured with Opti-MEM I (Thermo Fisher Scientific) for 48 h. For plasmid transfection, 7.0 × 10^6^ cells or 2.5 × 10^6^ cells were cultured for 24 h on a 15- or 10-cm dish, respectively, and then plasmids were introduced as described below.

### Plasmid construction

The primers used for plasmid construction are listed in Table S4. For construction of the expression plasmid of 3 × HA-tagged human SPP, the full-length cDNA of SPP was amplified by PCR using primers #1 to #2 from the cDNA library prepared from the total RNA of HEK293 cells. The PCR products were inserted into the BamHI-EcoRI site of pcDNA6/myc-His A vector using NEBuilder HiFi DNA Assembly Master Mix (New England Biolabs) to generate the pcDNA6/myc-His A SPP-mycHis plasmid. The constructed plasmid was then used as a template to amplify the SPP cDNA using primers #3 to #4, and the PCR product was inserted into the XhoI-MluI site of pME-3HA vector using NEBuilder HiFi DNA Assembly Master Mix. The expression plasmid of 3 × HA-tagged human SPPL3 was previously constructed (10).

The expression plasmid of myc/6 × His-tagged human FUT8 was previously constructed (31). For the construction of expression plasmids of FUT8Δlinker and FUT8 GS linker, two DNA fragments were amplified by PCR: one fragment corresponding to the 1st to 20st nucleotides of FUT8 cDNA (A in the initiation codon ATG is the first nucleotide) using primers #5 to #6 or #7 to #8, and the other fragment corresponding to the 235th to 1725th nucleotides of FUT8 using primers #5 to #9 or #7 to #10. These fragments were inserted into the BamHI-EcoRI site of the pcDNA6/mycHis A vector using NEBuilder HiFi DNA Assembly Master Mix. For the construction of expression plasmids of FUT8 point mutants, site-directed mutation was introduced using the QuikChange Lightning Site-Directed Mutagenesis Kit (Agilent Technology) with the pcDNA6/mycHis A FUT8 plasmid as a template. The following primers were used for point mutation: #11 to #12 for Y24A; #13 to #14 for I25A; #15 to #16 for G26A; #17 to #18 for G27A; #19 to #20 for H28A; and #21 to #22 for L29A.

For the construction of gene editing plasmids for the generation of SPP, SPPL2A, SPPL2B, and SPPL2C KO cells, pairs of oligonucleotides were inserted into the BbsI site of the px330-enhanced green fluorescent protein (EGFP) vector using the DNA Ligation Kit Ver. 2.1 (Takara). The oligonucleotide pairs used were as follows: SPPL2A, #23 to #24 and #25 to #26; SPPL2B, #27 to #28 and #29 to #30; SPPL2C, #31 to #32, and #33 to #34; and SPP, #35 to #36 and #37 to #138. The construction of px330-EGFP plasmid containing the sgRNA-targeting SPPL3 was described previously (10).

### Plasmid transfection

Cells on 10-cm dishes were transfected with 6 μg of plasmids using Lipofectamine 3000 Transfection Reagent (Thermo Fisher Scientific), followed by 48 h of culture. Subsequently, cells were harvested for Western blotting and *in vitro* glycosyltransferase activity assay. To collect proteins in the culture medium for glycosyltransferase activity assays or Western blotting, the culture medium was removed 24 h after transfection, and the cells were washed three times with PBS before replacing the medium with Opti-MEM I (Thermo Fisher Scientific). After an additional 48 h of culture, the cultured media were collected for experiments. To purify secreted FUT8 for *N*-terminal sequence analysis and to express 3 × HA-tagged SPP and SPPL3, HEK293 cells seeded on 15- or 10-cm dishes were transfected with plasmid using PEI MAX (Polysciences). Six hours later, the culture medium was removed, and the cells were washed three times with PBS. Subsequently, Opti-MEM I was added, and cells were cultured for 48 h before harvesting the culture media for further experiments. For immunofluorescence staining, cells cultured on a 6-cm dish were transfected with plasmids using Lipofectamine 3000 Transfection Reagent, and the cells were incubated at 37 °C under 5% CO_2_ conditions for 24 h. Subsequently, the cells were seeded on a 2-well glass-based chamber slide and incubated for 24 h.

### Establishment of SPP family KO cells

To generate HEK293 SPP KO, SPPL2A KO, SPPL2B KO, and SPPL2C KO cells, HEK293 cells were transfected with the plasmids expressing two different sgRNAs targeting each gene. Forty-eight hours after transfection, cells were harvested and Cas9-expressing cells were sorted based on EGFP expression using a FACSMelody Cell Sorter (BD Biosciences). Sorted cells were seeded onto 3.5-cm dishes and cultured for 48 h, followed by limiting dilution in 96-well plates to obtain single-cell clones. The genotypes were examined by PCR using the following primer pairs: SPP, #39 to #40; SPPL2A, #41 to #42; SPPL2B, #43 to #44; and SPPL2C, #45 to #46, and by sequencing. Generation of SPPL3 KO cells was previously reported (10). To generate HEK293 SPP/SPPL3 DKO cells, plasmids expressing sgRNAs targeting SPPL3 (10) were transfected into HEK293 SPP KO cells, and those targeting SPP were transfected into HEK293 SPPL3 KO cells. Single-cell clones were then isolated following the same procedure as for single KOs. Genotypes of the SPPL3 gene were confirmed by PCR using primers #47 to #48.

### Real-time PCR

Total RNA was prepared from HEK293 WT and various KO cells using TRI Reagent (Molecular Research Center), in accordance with the manufacturer’s protocol. Subsequently, cDNA was synthesized from total RNA using SuperScript IV First-Strand System (Thermo Fisher Scientific). The resulting cDNA was utilized for both real-time PCR analysis and plasmid construction. For real-time PCR, the target cDNAs were amplified using TaqMan Gene Expression Master Mix (Applied Biosystems) with the primers and probes listed below, and fluorescence signals were detected with CFX Connect Real-Time PCR Detection System (Bio-Rad). The expression levels of each mRNA were normalized to that of GAPDH. The following primers and probes were purchased from Applied Biosystems: *GAPDH*, Hs99999905_m1; and *FUT8*, Hs00189535_m1.

### Preparation of proteins from cell lysate

Cells were suspended in a lysis buffer consisting of Tris-buffered saline (TBS) containing 1% Triton X-100 and a protease inhibitor cocktail (FUJIFILM), and subsequently sonicated. The lysates were then centrifuged at 21,500×*g* for 20 min, and protein concentrations in the resulting supernatants were determined using Pierce bicinchoninic acid Protein Assay Kit (Thermo Fisher Scientific). These lysates were used for assays to measure glycosyltransferase activity. For SDS-PAGE analysis, lysates were mixed with Laemmli SDS sample buffer and heated at 95 °C for 5 min.

### Preparation of proteins from medium

For the preparation of proteins and secreted FUT8 from the culture medium, the cultured medium was applied to an Amicon Ultra Centrifugal Filter 10 kDa MWCO (Merck Millipore) and centrifuged at 3900×*g* for 30 min. The flow-through was discarded, and the sample was centrifuged again at 3900×*g* for 30 min. The concentrated fraction was collected and used for the measurement of secreted glycosyltransferase activity. To separate membrane components, including sEVs, from soluble components, the concentrated sample was subjected to ultracentrifugation at 100,000×*g* for 60 min. The supernatant (soluble fraction; Sup) and the pellet (membrane fraction; sEVs) were collected. The pellet was dissolved in lysis buffer (TBS containing 1% Triton X-100 and a protease inhibitor mixture; FUJIFILM) by sonication. The soluble and membrane fractions were used for glycosyltransferase activity assays. For SDS-PAGE analysis, the samples were mixed with Laemmli SDS sample buffer and heated at 95 °C for 5 min. For samples expressing 3 × HA-tagged SPP or SPPL3, Laemmli SDS sample buffer was added, and the samples were incubated on ice for 30 min instead of boiling before analysis. For glycoproteomics, the cultured medium was collected and ultracentrifuged at 100,000×*g* for 20 min at 4 °C. The supernatants were collected and mixed with a 1/30 volume of 5 M NaCl and 1/2.5 volume of ethanol, followed by incubation at −80 °C for 10 min. The mixture was centrifuged at 12,000×*g* for 30 min at 4 °C, and the precipitates were washed with 70% ethanol. The precipitates were then used for glycoproteomics.

### *In vitro* glycosyltransferase activity assays

The enzymatic activity of FUT8 was measured in accordance with a previously described method (25, 26) with slight modifications. Samples prepared from cell lysates or culture supernatants were incubated with 1 mM GDP-fucose and 10 μM fluorescently labeled acceptor substrate (GnGn-bi-Asn-PNSNB [N-(2-(2-pyridylamino)ethyl) succinamic acid 5-norbornene-2,3-dicarboxyimide ester] (43) or dansyl-labeled asialo-agalacto-biantennary glycan) in a total volume of 10 μl of FUT8 reaction buffer (100 mM MES-NaOH, pH 7.0, 200 mM GlcNAc, 0.5% Triton X-100, 1 mg/ml bovine serum albumin [BSA]) at 37 °C. For the measurement of GnT-I enzymatic activity, samples were mixed with 20 mM UDP-GlcNAc and 10 μM fluorescently labeled acceptor substrate (GlyTech, GT_25203) in 10 μl of GnT-I reaction buffer (125 mM MES-NaOH, pH 6.2, 10 mM MnCl_2_, 200 mM GlcNAc, 0.5% Triton X-100, 1 mg/ml BSA), and incubated at 37 °C. For the measurement of GnT-II activity, samples were mixed with 20 mM UDP-GlcNAc and 10 μM fluorescently labeled acceptor substrate (GlyTech, GT_25185) in 10 μl of the GnT-I reaction buffer, and incubated at 37 °C. For the measurement of GnT-III and GnT-V activities, samples were mixed with 20 mM UDP-GlcNAc and 10 μM fluorescently labeled acceptor substrate (GnGnbi-PA) (44) in 10 μl of the GnT-I reaction buffer, and incubated at 37 °C. For the measurement of B4GALT1 activity, samples were mixed with 1 mM UDP-Gal and 10 μM fluorescently labeled acceptor substrate (GnGnbi-PA) in 10 μl of reaction buffer (20 mM Tris–HCl, pH 8.0, 10 mM MnCl_2_, 0.5% Triton X-100), and incubated at 37 °C. For the measurement of ST6GAL1 activity, samples were mixed with 2 mM CMP-Neu5Ac and 2.5 μM fluorescently labeled acceptor substrate (GGnGGnbi-PA) (45) in 10 μl of the GnT-I reaction buffer, and incubated at 37 °C.

After incubation, the reactions were terminated by boiling at 95 °C for 5 min, followed by the addition of 40 μl of water and centrifugation at 21,500×*g* for 5 min. For HPLC analysis, 10 μl of the supernatants from FUT8, GnT-II, GnT-III, GnT-V, B4GALT1, and ST6GAL1 reactions were injected into an HPLC system equipped with an ODS column (GL Sciences, 4.6 × 250 mm). For the GnT-I reaction, 10 μl of the reaction supernatant was injected into an HPLC system equipped with a TSK gel Amide-80 3 μm column (Tosoh, 4.6 × 150 mm) to detect fluorescently labeled substrates and products. For HPLC analysis, the following mobile phases were used: for GnGnbi-Asn-PNSNB and FUT8 products, 80% buffer A (20 mM ammonium acetate, pH 4.0) and 20% buffer B (buffer A with 1% 1-butanol); for dansyl-labeled asialo-agalacto-biantennary glycan and FUT8 products, 80% buffer A containing 5 μM citric acid and 20% acetonitrile; for GnT-I substrates and products, starting with 80% acetonitrile and 20% buffer A (20 mM ammonium acetate, pH 4.0), a linear gradient was applied to reach 50% buffer A over 20 min; for GnT-II, GnT-III, GnT-V, and ST6GAL1 substrates and products, 80% buffer A (20 mM ammonium acetate, pH 4.0) and 20% buffer B (buffer A with 1% 1-butanol); and for B4GALT1 substrates and products, 95% buffer A and 5% buffer B. The specific activity in cell lysates, culture media, and Sup was calculated by determining the amounts of the reaction product based on the peak areas in the HPLC chromatograms and by dividing these amounts by the reaction time and the levels of cellular proteins.

### Western blotting

Proteins were separated by SDS-PAGE using a 5% to 20% gradient gel. Subsequently, the separated proteins in the gel were transferred to a nitrocellulose membrane. The membranes were blocked in TBS containing 5% skim milk and 0.1% Tween-20, and then incubated with primary antibody overnight at 4 °C. After washing three times with TBS containing 0.1% Tween-20, the membrane was incubated with HRP-conjugated secondary antibody at room temperature for 1 h. Following three washes with TBS containing 0.1% Tween-20, the membrane was incubated with either Western Lightning Plus-ECL (PerkinElmer) or SuperSignal West Femto Maximum Sensitivity Substrate (Thermo Fisher Scientific). The chemiluminescence signal was detected using FUSION-SOLO 7s (Vilber-Lourmat). The band intensity was quantified using ImageJ software.

### N-terminal sequence analysis of secreted FUT8

Secreted myc/6 × His-tagged FUT8 was purified from the culture media of HEK293 expressing myc/6 × His-tagged full-length FUT8 using Ni^2+^ beads. After SDS-PAGE, proteins were transferred to a polyvinylidene difluoride membrane and then stained with GelCode Blue Safe Protein Stain (Thermo Fisher Scientific). The FUT8 bands were excised, and the *N*-terminal sequence of the secreted FUT8 was determined by Edman degradation. Edman degradation and *N*-terminal sequencing were performed in accordance with the previously established method at the Institute for Protein Research, Osaka University (46). Briefly, the excised protein bands were subjected to a gas-phase protein sequencer, PPSQ-53A gradient system (Shimadzu), with eight cycles of a standard Edman degradation program. The reaction products, phenylthiohydantoin amino acids, were automatically separated on an octadecyl HPLC column, and then identified and quantified from the elution profile using a standard phenylthiohydantoin amino acid mixture (FUJIFILM Wako).

### Structure representation

The simplified α-helix models were created using the build tool in PyMOL (The PyMOL Molecular Graphics System, Version 3.0 Schrödinger, LLC.). Helix lengths corresponded to the designated amino acid sequences.

### Immunofluorescence staining

Cells cultured on 2-well glass chamber slides were washed three times with PBS following removal of the culture medium. Cells were then fixed with PBS containing 4% paraformaldehyde for 15 min at room temperature. After washing three times with PBS, cells were permeabilized and blocked with blocking solution (PBS containing 1% BSA and 0.1% Triton X-100) for 15 min at room temperature. Subsequently, the blocking solution was removed, and cells were incubated with primary antibody diluted in the blocking solution for 1 h at room temperature. Following washing three times with PBS, cells were incubated with secondary antibody and 4′,6-diamidino-2-phenylindole diluted in the blocking solution for 30 min at room temperature. After washing four times with PBS, the chamber slides were dried and then the chamber was removed. Next, ProLong Diamond Antifade Mountant (Invitrogen) was applied, and cover glass was placed on the slide glass. Fluorescence was visualized using a BZ-X800 all-in-one microscope (KEYENCE).

### Evaluation of signal sequence of FUT8

Signal peptide prediction was performed using SignalP-6.0 (DTU Health Tech). The 1st to 40th amino acid residues of human calnexin, FUT8 WT, or FUT8 point mutants were analyzed, with “Eukarya” being selected as the organism group, “long” as the output format, and “fast” as the model mode.

### FACS

Cells were suspended with PBS and then centrifuged at 1500×*g* for 3 min, and the supernatant was discarded. Subsequently, the cells were incubated with biotinylated PhoSL diluted in fluorescence activated cell sorting (FACS) buffer (1% BSA in PBS) on ice for 1 h. After centrifugation at 1500×*g* for 3 min and removal of the supernatant, cells were washed three times with FACS buffer. Cells were then suspended with Streptavidin-Alexa Fluor 488 (Invitrogen, S11223) diluted in FACS buffer and incubated on ice for 30 min. Following centrifugation at 1500×*g* for 3 min and removal of the supernatant, cells were washed four times with PBS and then fluorescence signals were detected using FACSMelody Cell Sorter (BD Biosciences). Data were analyzed using FlowJo software (BD Biosciences).

### Protein digestion and glycopeptide enrichment

Protein extracts (50 μg) were reduced using 10 mM DTT (30 min, 30 °C) and alkylated using 20 mM iodoacetamide (final concentration, 30 min, in the dark, 20 °C). Alkylation reactions were quenched using 20 mM DTT. Samples were digested using sequencing-grade porcine trypsin (1:50, w/w; 12 h, 37 °C, Promega). Proteolysis was stopped using 1% (v/v) TFA (final concentration). Peptides were desalted on primed Oligo R3 reversed-phase SPE microcolumns, aliquoted, and dried. A small aliquot containing 5 μg of desalted peptides was used directly for LC-MS/MS analysis (unenriched peptide fractions). Dried peptide mixtures were reconstituted in 50 μl of 80% acetonitrile in 1% (both v/v) TFA and loaded onto primed custom-made hydrophilic interaction liquid chromatography SPE micro-columns packed with zwitterionic ZIC-hydrophilic interaction liquid chromatography resin (10 μm particle size, 200 Å pore size, kindly provided by Merck Millipore) onto supporting C8 disks (Empore) in p10 pipette tips, as described previously (47). The retained glycopeptides were eluted over three rounds with 0.1% (v/v) TFA, 25 mM ammonium bicarbonate, and then 50% (v/v) acetonitrile, and the eluted fractions were pooled. The enriched glycopeptides were desalted on primed Oligo R3 reversed-phase SPE microcolumns, aliquoted, and dried.

### Glycopeptide and peptide profiling by reversed-phase LC-MS/MS

Unenriched peptides or enriched glycopeptides were loaded on a PepMap Neo Nano Trap Cartridge (5 mm × 300 μm inner diameter, Thermo Fisher Scientific) and separated on an analytical column (Aurora Ultimate; 25 cm × 75 μm, 1.7 μm ID, IonOpticks) at 300 nl/min provided by a Vanquish Neo UHPLC System (Thermo Fisher Scientific). The mobile phases were 99.9% acetonitrile in 0.1% (both v/v) formic acid (solvent B) and 0.1% (v/v) formic acid (solvent A). The gradient was 3% to 35% B over 90 min, 35% to 50% B over 8 min, 50% to 90% B over 2 min, and 10 min at 95% B. The nanoLC was connected to an Orbitrap Exploris 240 mass spectrometer (Thermo Fisher Scientific) operating in positive ion polarity mode.

For the enriched glycopeptide fraction, the Orbitrap was used to acquire full MS1 scans (*m/z* 500–2,000, AGC: standard, 100 ms maximum accumulation, 120,000 FWHM resolution at *m/z* 200). Employing data-dependent acquisition within a cycle time of 3 s, the most abundant precursor ions from each MS full scan were isolated and fragmented utilizing stepped higher-energy collision-induced dissociation (HCD, %) 20, 30, and 40. Only multicharged precursors (Z ≥ 2) were selected for fragmentation. Fragment spectra were acquired in the Orbitrap (15,000 resolution, AGC: standard, 200 ms maximum accumulation, *m/z* 1.6 precursor isolation window, and 20 s dynamic exclusion after a single isolation/fragmentation of a given precursor).

For the unenriched peptide fraction, the Orbitrap was used to acquire full MS1 scans (*m/z* 380–1,800, AGC: standard, 100 ms maximum accumulation, 120,000 FWHM resolution at *m/z* 200). Employing data-dependent acquisition within a cycle time of 3 s, the most abundant precursor ions from each MS full scan were isolated and fragmented utilizing HCD of 30%. Only multicharged precursors (Z ≥ 2) were selected for fragmentation. Fragment spectra were acquired in the Orbitrap (15,000 resolution, AGC: standard, maximum infection time set to “auto,” *m/z* 1.4 precursor isolation window, and 20 s dynamic exclusion after a single isolation/fragmentation of a given precursor). Each biological replicate was analyzed in a single technical replicate.

### Glycopeptide identification and quantification

Glycopeptides were identified and quantified from the HCD-MS/MS data using Byonic v5.4.10 (Protein Metrics) operated as a node in Proteome Discoverer v.3.0. Search parameters included the use of i) a human proteome database (UniProtKB, 20,419 entries, downloaded March 2023), ii) an *N*-glycan database comprising the 183 human *N*-glycan components, and iii) a search strategy permitting up to one *N*-glycan per peptide as a “rare” variable modification, fully-trypsin cleavage patterns with a maximum of two missed tryptic cleavages per peptide, up to 10/20 ppm mass tolerance of precursor/product ions from expected values, and up to one Met oxidation (+15.994 Da) per peptide (variable “common” modification), and by using monoisotopic correction (error check = +/− floor [mass in Da/4000]), and a decoy and a default contaminant database available in Byonic. Glycopeptide identifications were filtered to false discovery rate (FDR) < 1% (high confidence as determined using Proteome Discoverer 3.0). Glycopeptides identified with lower confidence and those found in the decoy and contaminant database were excluded. Quantitation of glycopeptides was based on the area under the curve of extracted ion chromatograms of precursor ions as determined by the Minora Feature Detector with the minimum trace length set to 5 and maximum delta retention time of isotope pattern multiplets of 0.2 min within Proteome Discoverer v2.5. The feature mapper tool was enabled to allow chromatographic alignment of signals and extraction of precursor area based on a matched retention time and *m/z* within a time and mass tolerance window determined automatically by the software. Minimum S/N thresholds were enabled.

Glycopeptides containing diagnostic ions for core fucosylation were identified using Glyco-decipher v1.0.5 (48) applying the following search parameters: a human proteome database (UniProtKB, 20,419 entries, downloaded March 2023), fully trypsin cleavage patterns with a maximum of three missed tryptic cleavages per peptide, up to 10/20 ppm mass tolerance of precursor/product ions from expected values, met oxidation (+15.994 Da) as variable modification, and cys carbamidomethylation (+57.021 Da) as fixed modification. Spectrum expansion was allowed.

### Peptide identification and quantification

For protein identification and quantification, the raw files from the unenriched fraction were imported into MaxQuant v1.6.10.4 (49). The Andromeda search engine was used to search the HCD-MS/MS data against the reviewed UniProtKB Human Protein Database (released December 2019; 20,364 entries) with a precursor and product ion mass tolerance of 4.5 ppm and 20 ppm, respectively. Carbamidomethylation of cysteine (57.021 Da) was set as a fixed modification. Oxidation of methionine (15.994 Da) and protein N-terminal acetylation (42.010 Da) were selected as variable modifications. All identifications were filtered to <1% protein FDR using a conventional decoy approach. For label-free area under the curve–based quantification, the “match between runs” feature of MaxQuant was enabled with a 0.7 min match time window and 20 min alignment time window. Protein abundance was calculated based on the normalized protein intensity (LFQ intensity).

### Statistical analysis

Statistical analyses were performed using GraphPad Prism 8 (GraphPad Software). The individual data points in the graphs are biological replicates. Statistical significance was assessed using one-way ANOVA with *post hoc* Dunnett’s multiple-comparison test or two-way ANOVA with *post hoc* Tukey’s multiple-comparison test, with significance levels indicated as *p* < 0.05 (∗), *p* < 0.01 (∗∗), *p* < 0.001 (∗∗∗), and *p* < 0.0001 (∗∗∗∗). Differential proteomics analyses were performed using Perseus (v2.0.9.0). Statistical significance was determined by one-way ANOVA followed by Benjamini–Hochberg correction for multiple testing, with an FDR threshold of <0.05. Heat maps and hierarchical clustering analyses were generated in Perseus (v2.0.9.0) using Euclidean distance and average linkage clustering.

## Data availability

The glycoproteomics and proteomics LC-MS/MS raw data have been deposited to GlycoPost (50) with the identifier GPST000621.

## Supporting information

This article contains supporting information.

## Conflict of interest

One of the authors (Y. K.) is an Editorial Board Member for this journal and was not involved in the editorial review or the decision to publish this article. The other authors declare that they have no conflicts of interest with the contents of this article.
