# Supplementary material for: Signal peptide peptidase– and SPP-like 3–dependent shedding of α1,6-fucosyltransferase differentially affects core fucosylation
Source: J Biol Chem. 2026 Jan 29;302(3):111209. doi: 10.1016/j.jbc.2026.111209 (PMC12927314; doi:10.1016/j.jbc.2026.111209)
Supplement: Supplementary Material [file mmc1.pdf]

Supporting Information

# Signal peptide peptidase (SPP)- and SPP-like 3 (SPPL3)-dependent shedding of α1,6-fucosyltransferase (FUT8) differentially affects core fucosylation

**Seita Tomida, Rebeca Kawahara, Kristina Mae Bienes, Yuko Tokoro, Takahiro Yamasaki, Yasuhiko Kizuka***

*Correspondence: Yasuhiko Kizuka, Ph.D., kizuka.yasuhiko.k8@f.gifu-u.ac.jp.

This Supporting information includes:

Figs. S1–S6 (included in this PDF)

Tables S1-S3 (separate file)

Table S4 (included in this PDF)

Figure S1

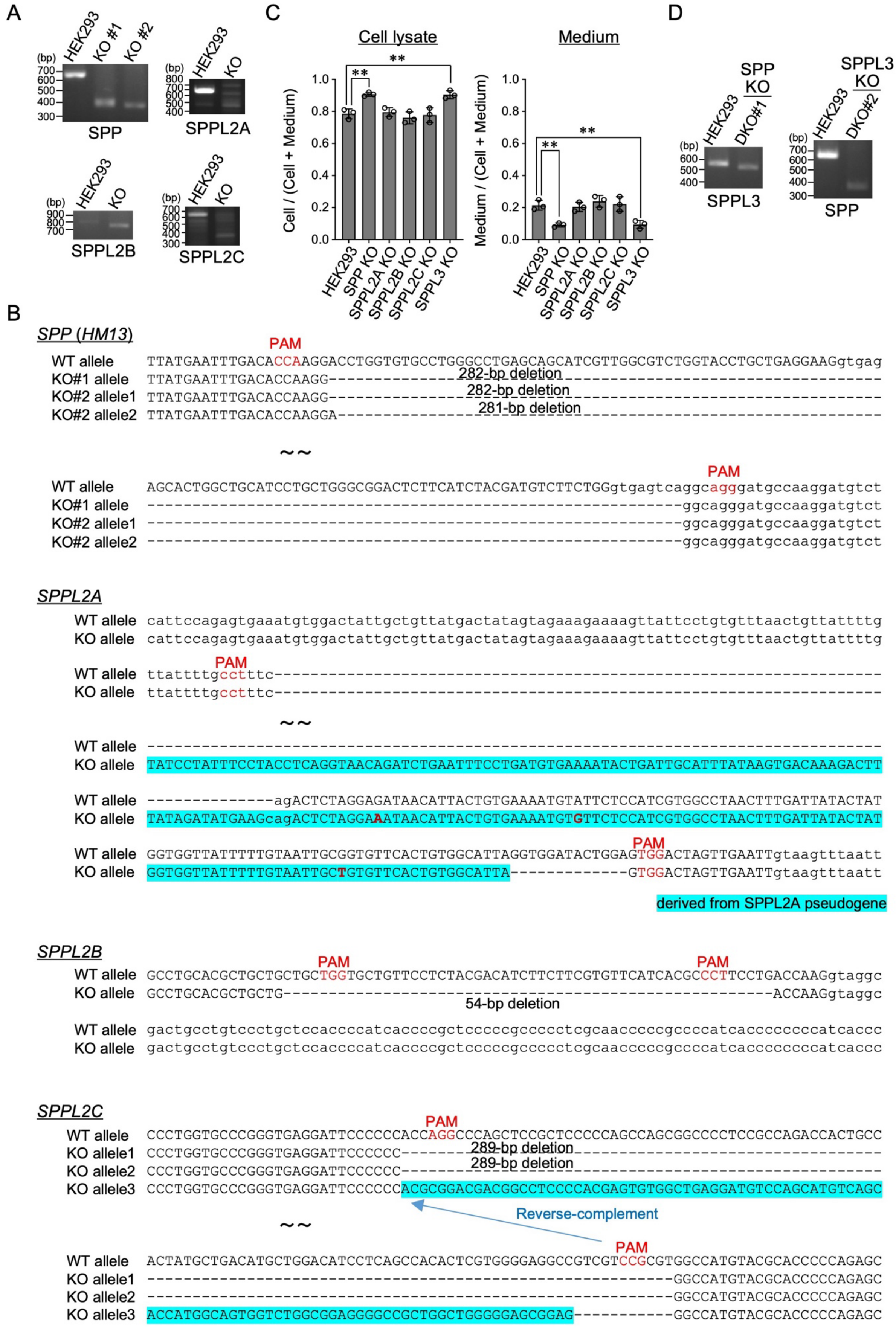

A
(bp)
700
600
500
400
300
HEK293
KO #1
KO #2
SPP
HEK293
KO
800
700
600
500
400
SPPL2A
HEK293
KO
900
800
700
SPPL2B
HEK293
KO
700
600
500
400
300
SPPL2C
C
Cell lysate
Medium
**
Cell / (Cell + Medium)
Medium / (Cell + Medium)
0.0
0.2
0.4
0.6
0.8
1.0
HEK293
SPP KO
SPPL2A KO
SPPL2B KO
SPPL2C KO
SPPL3 KO
D
SPP KO
HEK293
DKO#1
600
500
400
SPPL3
SPPL3 KO
HEK293
DKO#2
700
600
500
400
300
SPP
B
SPP (HM13)
PAM
WT allele TTATGAATTTGACACCAAGGACCTGGTGTGCCTGGGCCTGAGCAGCATCGTTGGCGTCTGGTACCTGCTGAGGAAGgtgag
KO#1 allele TTATGAATTTGACACCAAGG
282-bp deletion
KO#2 allele1 TTATGAATTTGACACCAAGG
282-bp deletion
KO#2 allele2 TTATGAATTTGACACCAAGGA
281-bp deletion
~~
PAM
WT allele AGCACTGGCTGCATCCTGCTGGGCGGACTCTTCATCTACGATGTCTTCTGGgtgagtcaggcagggatgccaaggatgtct
KO#1 allele ggcagggatgccaaggatgtct
KO#2 allele1 ggcagggatgccaaggatgtct
KO#2 allele2 ggcagggatgccaaggatgtct
SPPL2A
WT allele cattccagagtgaaatgtggactattgctgttatgactatagtagaaagaaaagttattcctgtgtttaactgttattttg
KO allele cattccagagtgaaatgtggactattgctgttatgactatagtagaaagaaaagttattcctgtgtttaactgttattttg
PAM
WT allele ttattttgcctttc
KO allele ttattttgcctttc
~~
WT allele
KO allele TATCCTATTTCCTACCTCAGGTAACAGATCTGAATTTCCTGATGTGAAAATACTGATTGCATTTATAAGTGACAAAGACTT
WT allele agACTCTAGGAGATAACATTACTGTGAAAATGTATTCTCCATCGTGGCCTAACTTTGATTATACTAT
KO allele TATAGATATGAAGcagACTCTAGGAAATAACATTACTGTGAAAATGTGTTCTCCATCGTGGCCTAACTTTGATTATACTAT
PAM
WT allele GGTGGTTATTTTTGTAATTGCGGTGTTCACTGTGGCATTAGGTGGATACTGGAGTGGACTAGTTGAATTgtaagtttaatt
KO allele GGTGGTTATTTTTGTAATTGCTGTGTTCACTGTGGCATTA GTGGACTAGTTGAATTgtaagtttaatt
derived from SPPL2A pseudogene
SPPL2B
PAM
PAM
WT allele GCCTGCACGCTGCTGCTGCTGGTGCTGTTCCTCTACGACATCTTCTTCGTGTTCATCACGCCCTTCCTGACCAAGgtaggc
KO allele GCCTGCACGCTGCTG ACCAAGgtaggc
54-bp deletion
WT allele gactgcctgtccctgctccaccccatcaccccgctcccccgccccctcgcaaccccgccccatcacccccccccatcaccc
KO allele gactgcctgtccctgctccaccccatcaccccgctcccccgccccctcgcaaccccgccccatcacccccccccatcaccc
SPPL2C
PAM
WT allele CCCTGGTGCCCGGGTGAGGATTCCCCCCACCAGGCCCAGCTCCGCTCCCCCAGCCAGCGGCCCCTCCGCCAGACCACTGCC
KO allele1 CCCTGGTGCCCGGGTGAGGATTCCCCCC
289-bp deletion
KO allele2 CCCTGGTGCCCGGGTGAGGATTCCCCCC
289-bp deletion
KO allele3 CCCTGGTGCCCGGGTGAGGATTCCCCCCACGCGGACGACGGCCTCCCCACGAGTGTGGCTGAGGATGTCCAGCATGTCAGC
Reverse-complement
~~
PAM
WT allele ACTATGCTGACATGCTGGACATCCTCAGCCACACTCGTGGGGAGGCCGTCGTCCGCGTGGCCATGTACGCACCCCCAGAGC
KO allele1 GGCCATGTACGCACCCCCAGAGC
KO allele2 GGCCATGTACGCACCCCCAGAGC
KO allele3 ACCATGGCAGTGGTCTGGCGGAGGGGCCGCTGGCTGGGGGAGCGGAG GGCCATGTACGCACCCCCAGAGC

**Figure S1. Validation of SPP/SPPL KO cells.** ***A***, Genomic DNAs from HEK293 SPP KO cells, SPPL2A KO cells, SPPL2B KO cells, and SPPL2C KO cells was extracted, and subjected to genotyping PCR was performed. ***B***, Genomic DNA from HEK293 SPP KO, SPPL2A KO, SPPL2B KO, and SPPL2C KO cells was analyzed by Sanger sequencing. ***C***, The ratios of FUT8 activity in cell lysates or culture medium were calculated by dividing the activity in the cell lysate or medium by the sum of the activity in the cell lysate and medium, based on the specific activity shown in Fig. 1B. (n = 3, mean ± SD, ∗∗: $p < 0.01$, Dunnett's multiple comparison test). ***D***, Genomic DNA from HEK293 WT cells, and SPP/SPPL3 DKO#1 and #2 cells was analyzed by PCR for genotyping.

Figure S2

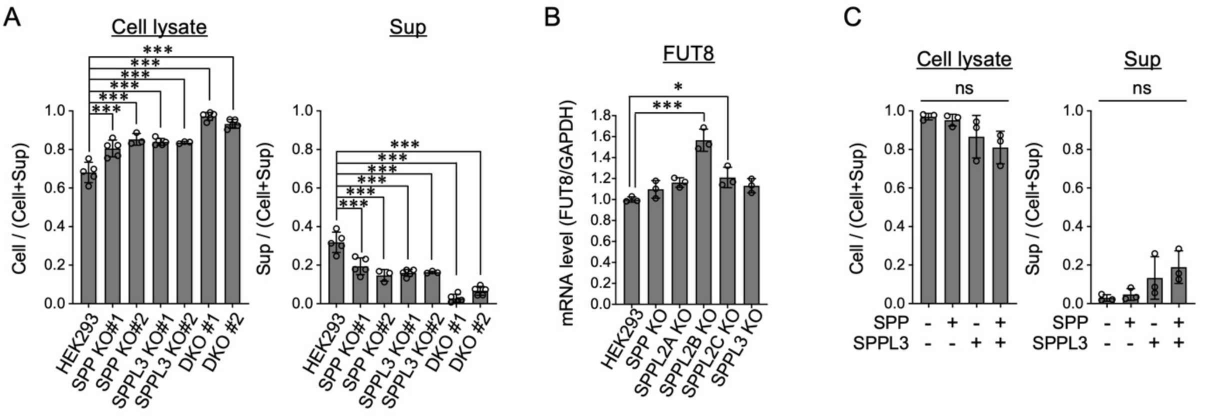


**Figure S2. Ratios of FUT8 activity in cell lysate and sup and mRNA levels of FUT8 in KO cells.** ***A***, The ratios of FUT8 activity in cell lysates or soluble fractions (Sup) were calculated by dividing the activity in the cell lysate or Sup by the sum of the activity in the cell lysate and Sup, based on the specific activity shown in Fig. 1C. (HEK293, SPP KO#1, SPPL3 KO#1, DKO#,1 and DKO#2: *n* = 5, SPP KO#2, SPPL3 KO#2: *n* = 3, mean ± SD, ns: not significant, ∗∗∗: p < 0.001, Dunnett's multiple comparison test). ***B***, The mRNA expression levels of FUT8 in HEK293 WT cells and SPP/SPPL single-KO cell lines were quantified by real-time PCR. The expression levels were normalized to that of GAPDH and are shown as values relative to that of HEK293 WT (*n* = 3, mean ± SD, ∗: p < 0.05, ∗∗: p < 0.01, ∗∗∗: p < 0.001, Dunnett's multiple comparison test). ***C***, The ratios of FUT8 activity in cell lysates or Sup based on the specific activity shown in Fig. 1H (*n* = 3, mean ± SD, ns: not significant, Dunnett's multiple comparison test).

# Figure S3

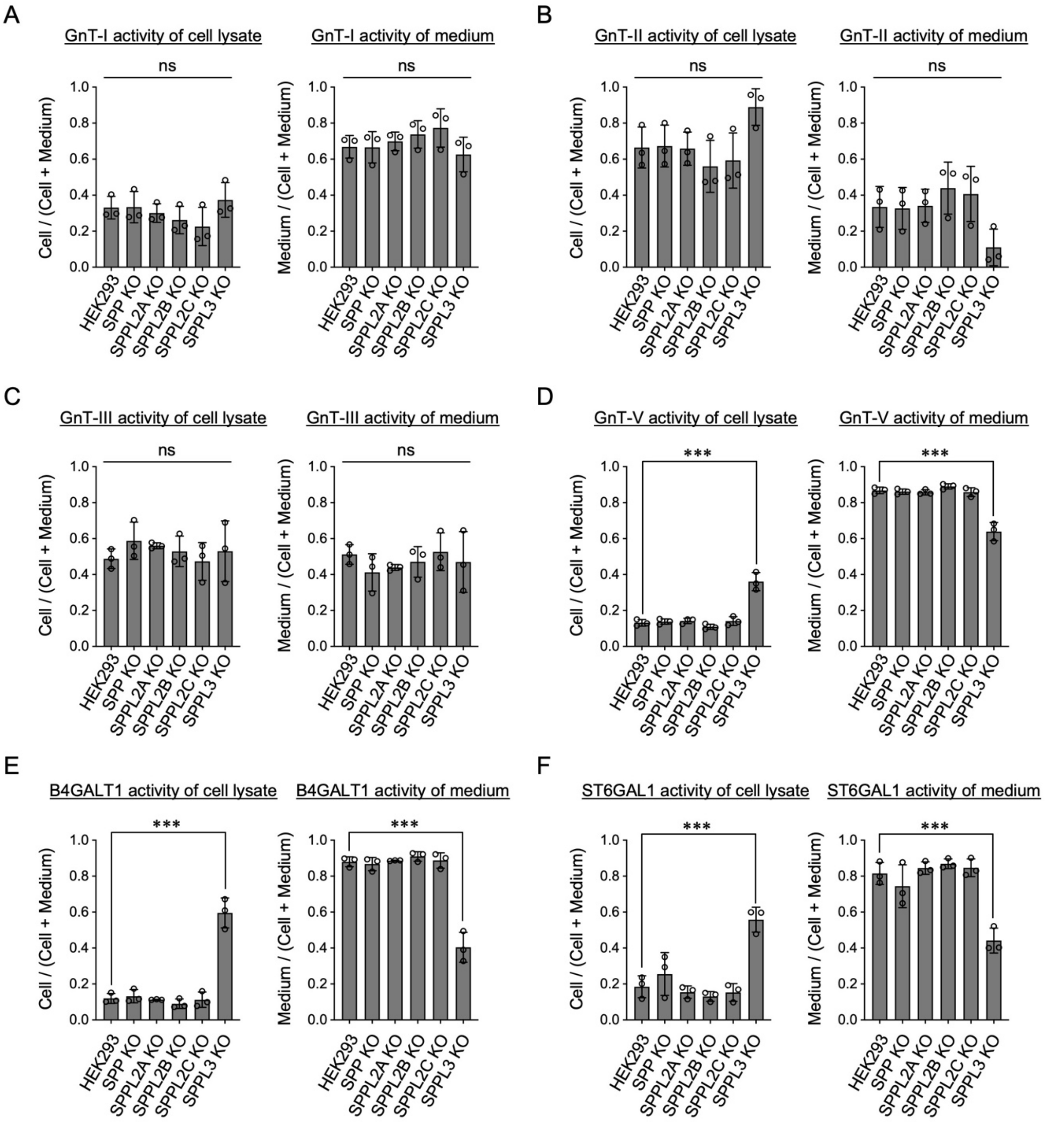


**Figure S3. Ratio of glycosyltransferase activity in cell lysate and medium.** The ratios were calculated based on the specific activity shown in Fig. 2. (n = 3, mean ± SD, ns: not significant, ∗∗∗: $p < 0.001$, Dunnett's multiple comparison test).

Figure S4

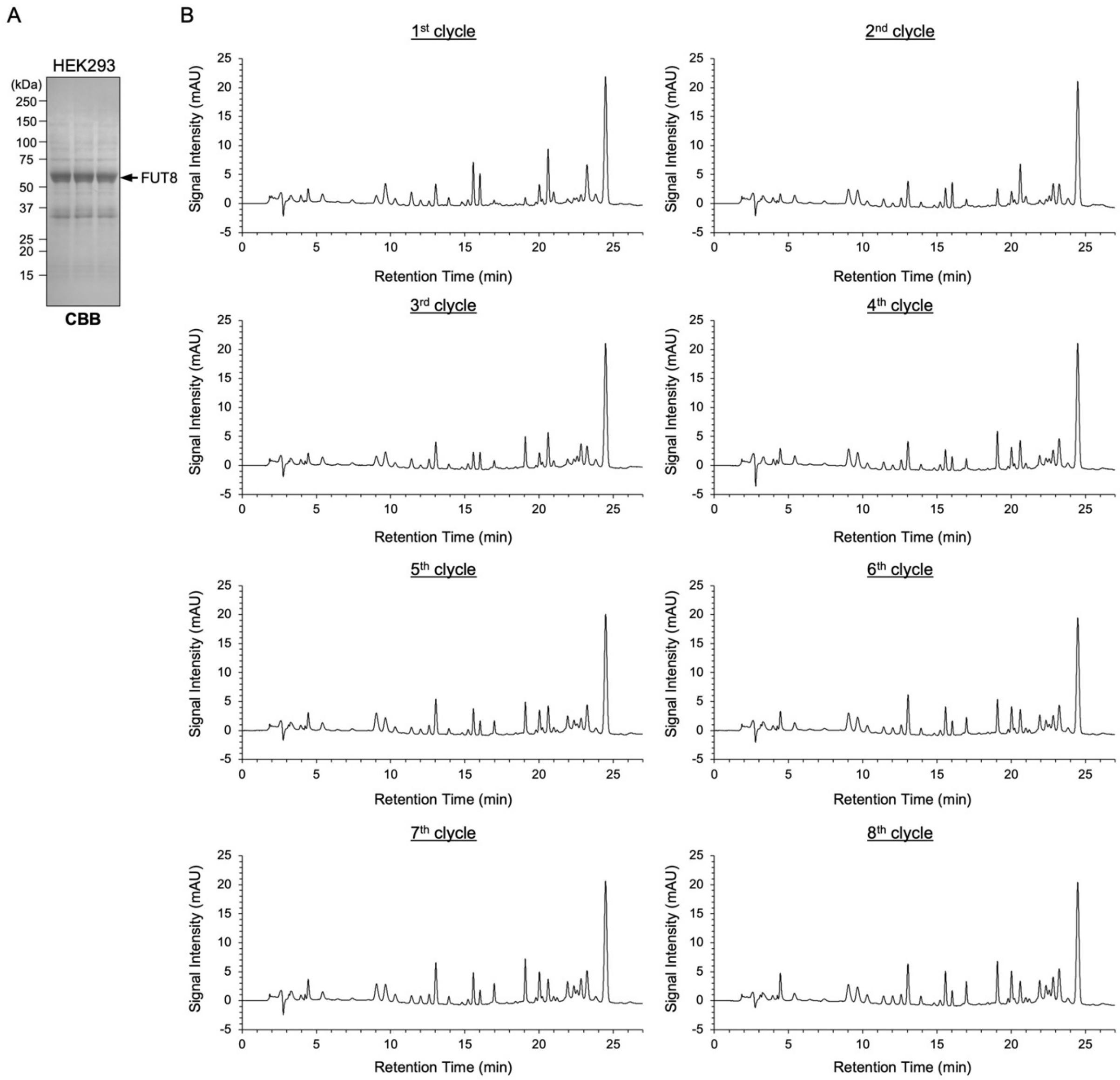


**Figure S4. *N*-terminal sequence analysis of secreted FUT8.** ***A***, Myc/His-tagged FUT8 was expressed in HEK293 cells and purified from the culture medium using $Ni^{2+}$-beads. Purified secreted FUT8 was subjected to SDS-PAGE, transferred to a PVDF membrane, and subjected to CBB staining. ***B***, The *N*-terminal sequence of secreted FUT8 was analyzed by Edman degradation. The chromatograms from the first to eighth cycles are shown.

# Figure S5

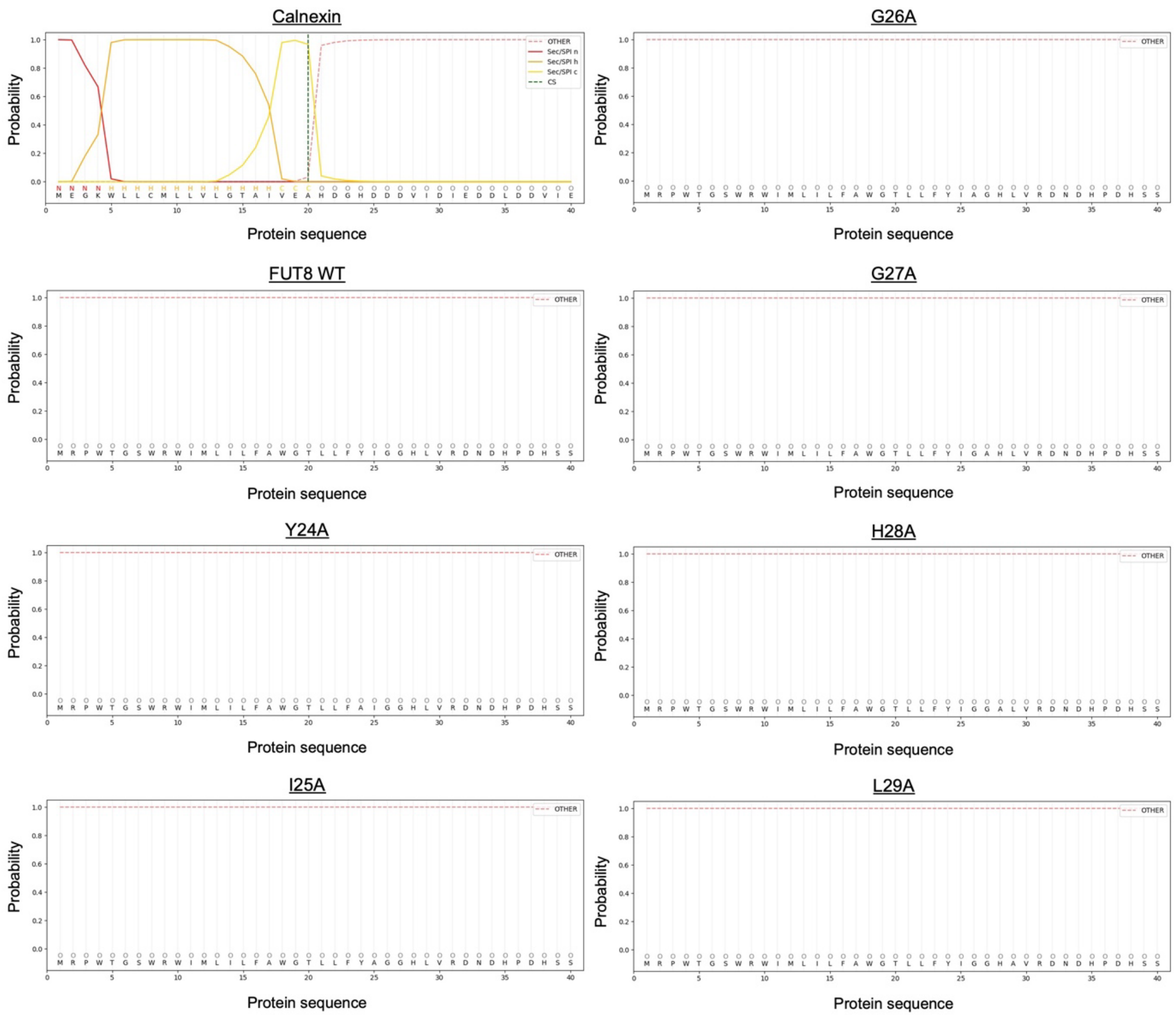


**Figure S5. Prediction of signal peptide properties of the transmembrane region of FUT8.** The 40 amino acid residues from the *N*-terminus of FUT8 and its transmembrane domain mutants were analyzed using SignalP 6.0 to evaluate the probability of a signal peptide. The *N*-terminal 40 amino acids of human calnexin were used as a control having an *N*-terminal signal peptide.

Figure S6

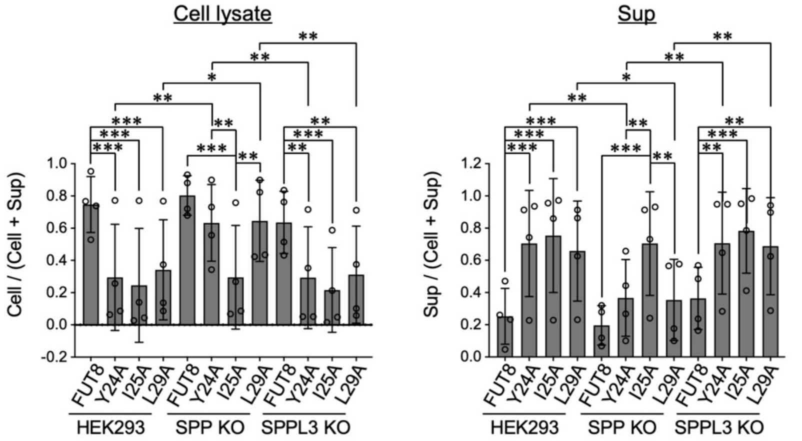


**Figure S6. Ratios of FUT8 mutant activities in cell lysate and Sup.** The ratios were calculated based on the specific activities shown in Fig. 4F. (n = 4, mean ± SD, ns: not significant, ∗: $p < 0.05$, ∗∗: $p < 0.01$, ∗∗∗: $p < 0.001$, two-way ANOVA with post-hoc Tukey's multiple comparison test).

# Table S4.

Oligonucleotides used in this study.

| No. | Name | Sequence |
|---|---|---|
| #1 | HM13_Fw | TTAAGCTTGGTACCGAGCTCGGATCCGCCACCATGGACTCGGCCCTCAGCGA |
| #2 | HM13_Rv | GCTGGATATCTGCAGAATTCTTTCTCTTTCTTCTCCAGCC |
| #3 | HM13-3HA_Fw | GAATTCCGGAATTTCCTCGAGGCCACCATGGACTCGGCCCT |
| #4 | HM13-3HA_Rv | ATAGGGGTATCCGCCACGCGTTTTCTCTTTCTTCTCCAGCC |
| #5 | FUT8 mycHis Fw | TTAAGCTTGGTACCGAGCTCGGATCCGCCACCATGCGGCCATGGAC |
| #6 | FUT8 D helix linker_Rv | ACGCGTACTCTTCCTATAGCGAGAGATTCGGCCATTCGCC |
| #7 | FUT8 mycHis Rv | GCTGGATATCTGCAGAATTCTTTCTCAGCCTCAGGATATG |
| #8 | FUT8 D helix linker_Fw | GGCGAATGGCCGAATCTCTCGCTATAGGAAGAGTACGCGT |
| #9 | FUT8 D helix GS linker_Rv | ACGCGTACTCTTCCTATAGCTGAGCCACCGCCTCCGAGAGATTCGGCCATTCGCC |
| #10 | FUT8 D helix GS linker_Fw | GGCGAATGGCCGAATCTCTCGGAGGCGGTGGCTCAGCTATAGGAAGAGTACGCGT |
| #11 | FUT8 Y24A_Fw | CCTGGGGGACCTTGCTGTTTGCAATAGGTGGTCACTTGGTACG |
| #12 | FUT8 Y24A_Rv | CGTACCAAGTGACCACCTATTGCAAACAGCAAGGTCCCCCAGG |
| #13 | FUT8 I25A_Fw | GGGGGACCTTGCTGTTTTATGCAGGTGGTCACTTGGTACGAGA |
| #14 | FUT8 I25A_Rv | TCTCGTACCAAGTGACCACCTGCATAAAACAGCAAGGTCCCCC |
| #15 | FUT8 G26A_Fw | GGACCTTGCTGTTTTATATAGCAGGTCACTTGGTACGAGATAA |
| #16 | FUT8 G26A_Rv | TTATCTCGTACCAAGTGACCTGCTATATAAAACAGCAAGGTCC |
| #17 | FUT8 G27A_Fw | CCTTGCTGTTTTATATAGGTGCACACTTGGTACGAGATAATGA |
| #18 | FUT8 G27A_Rv | TCATTATCTCGTACCAAGTGTGCACCTATATAAAACAGCAAGG |
| #19 | FUT8 H28A_Fw | TGCTGTTTTATATAGGTGGTGCATTGGTACGAGATAATGACCA |
| #20 | FUT8 H28A_Rv | TGGTCATTATCTCGTACCAATGCACCACCTATATAAAACAGCA |
| #21 | FUT8 L29A_Fw | TGTTTTATATAGGTGGTCACGCAGTACGAGATAATGACCATCC |
| #22 | FUT8 L29A_Rv | GGATGGTCATTATCTCGTACTGCGTGACCACCTATATAAAACA |
| #23 | SPPL2A KO1_Fw | CACCGTTATCTCCTAGAGTCTGAA |
| #24 | SPPL2A KO1_Rv | AAACTTCAGACTCTAGGAGATAAC |
| #25 | SPPL2A KO2_Fw | CACCGCATTAGGTGGATACTGGAG |
| #26 | SPPL2A KO2_Rv | AAACCTCCAGTATCCACCTAATGC |
| #27 | SPPL2B KO1_Fw | CACCGGCCTGCACGCTGCTGCTGC |
| #28 | SPPL2B KO1_Rv | AAACGCAGCAGCAGCGTGCAGGCC |
| #29 | SPPL2B KO2_Fw | CACCGTCGCCTACCTTGGTCAGGA |
| #30 | SPPL2B KO2_Rv | AAACTCCTGACCAAGGTAGGCGAC |
| #31 | SPPL2C KO1_Fw | CACCGGGTGAGGATTCCCCCCACC |
| #32 | SPPL2C KO1_Rv | AAACGGTGGGGGGAATCCTCACCC |
| #33 | SPPL2C KO2_Fw | CACCGGGGTGCGTACATGGCCACG |
| #34 | SPPL2C KO2_Rv | AAACCGTGGCCATGTACGCACCCC |
| #35 | HM13 KO1_Fw | CACCGCCCAGGCACACCAGGTCCT |
| #36 | HM13 KO1_Rv | AAACAGGACCTGGTGTGCCTGGGC |
| #37 | HM13 KO2_Fw | CACCGTCTTCTGGGTGAGTCAGGC |
| #38 | HM13 KO2_Rv | AAACGCCTGACTCACCCAGAAGAC |
| #39 | HM13 KO PCR_Fw | GTAAGTCTCTCTCTGGGCCTCC |
| #40 | HM13 KO PCR_Rv | AAACAGCTCTTCTCCGTCAG |
| #41 | SPPL2A KO PCR_Fw | TGGCACCAAGTGTCTCAAAG |
| #42 | SPPL2A KO PCR_Rv | AGGGTCTCTGATGTGATGTC |
| #43 | SPPL2B KO PCR_Fw | GAACTGGCCTCAGGTGGTAC |
| #44 | SPPL2B KO PCR_Rv | TTGAGTGAGGGAGCGTGGTG |
| #45 | SPPL2C KO PCR_Fw | GAAGAAGATGGCGTGCCTGG |
| #46 | SPPL2C KO PCR_Rv | AGCCGGTTGGCTTCGGTCAG |
| #47 | hSPPL3-geno-F | CAAGTCCAAACAGGCTGAAGAAG |
| #48 | hSPPL3-geno-R | ACTGCAGCGAATCTTGTCAGC |
| #49 | SPPL2B_seq1 | TGGACGTGACGCCGGTGATG |
